# Supplementary material for: Electricity-free hydrogen production from the air
Source: Nat Commun. 2026 Feb 4;17:1445. doi: 10.1038/s41467-025-67511-z (PMC12887035; doi:10.1038/s41467-025-67511-z)
Supplement: Supplementary file 1 — Supplementary Information [file 41467_2025_67511_MOESM1_ESM.pdf]

Supplementary Information

Electricity-Free Hydrogen Production from the Air

Qili Xu[1,2]†, Xiaoxue Yao[1]†, Hoi Ying Chung[2]†, Xiongyi Liang[3,4,5]†, Zhi Zhang[1], Zhenwen Zhang[1], Wai Kin Lo[1], Yijun Zeng[1], Xiao Cheng Zeng[3,6]*, Yun Hau Ng[2,7]*, Steven Wang[1]*

Affiliations:

[1]Department of Mechanical Engineering, City University of Hong Kong, Hong Kong, China
[2]School of Energy and Environment, City University of Hong Kong, Hong Kong, China
[3]Centre for Nature-inspiring Engineering, City University of Hong Kong, Hong Kong, China
[4]Department of Materials Science & Engineering, City University of Hong Kong, Hong Kong, China
[5]Shenzhen Research Institute, City University of Hong Kong, Shenzhen, China
[6]Chengdu Research Institute, City University of Hong Kong, Chengdu, China
[7]Hong Kong Institute for Clean Energy, City University of Hong Kong, Hong Kong, China
[8]Center for Renewable Energy and Storage Technologies (CREST), Clean Energy Research Platform (CERP), Chemical Engineering Program, Physical Science and Engineering Division, King Abdullah University of Science and Technology, Thuwal, Saudi Arabia

†Equal contribution

*Corresponding authors: xzeng26@cityu.edu.hk; yunhau.ng@kaust.edu.sa; steven.wang@city.edu.hk

Supplementary Information Contents:

Figs. S1 to S56

Note S1 to Note S3

Table S1 to S4

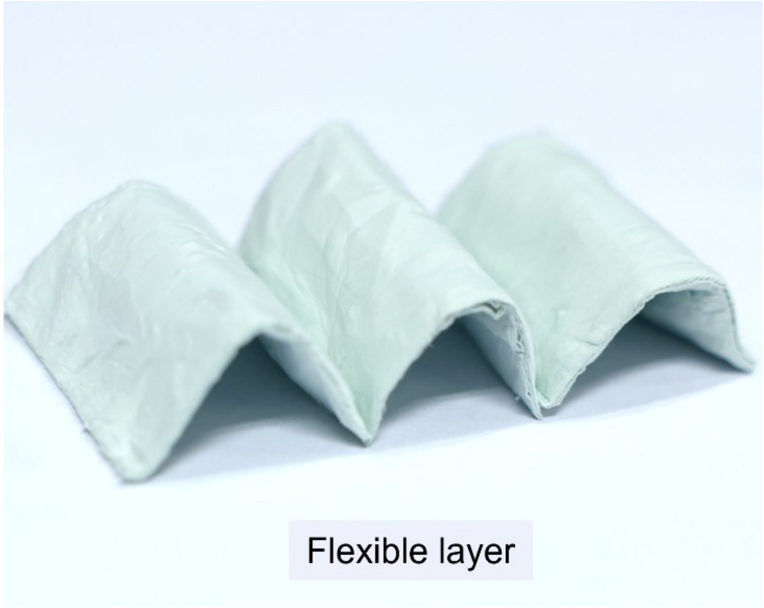

Fig. S1| Optical image of hygroscopic layer.

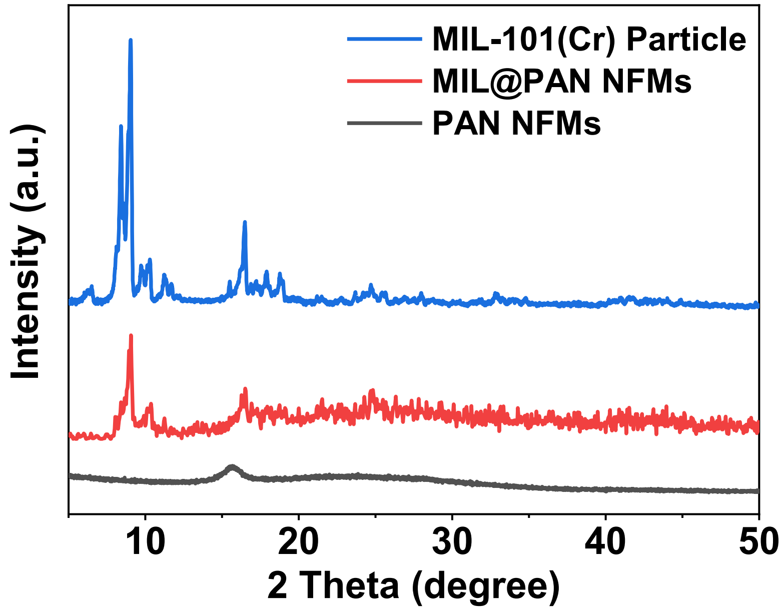

Fig. S2| X-ray diffraction (XRD) patterns of MIL-101(Cr) particles, MIL@PAN NFMs and PAN NFMs.

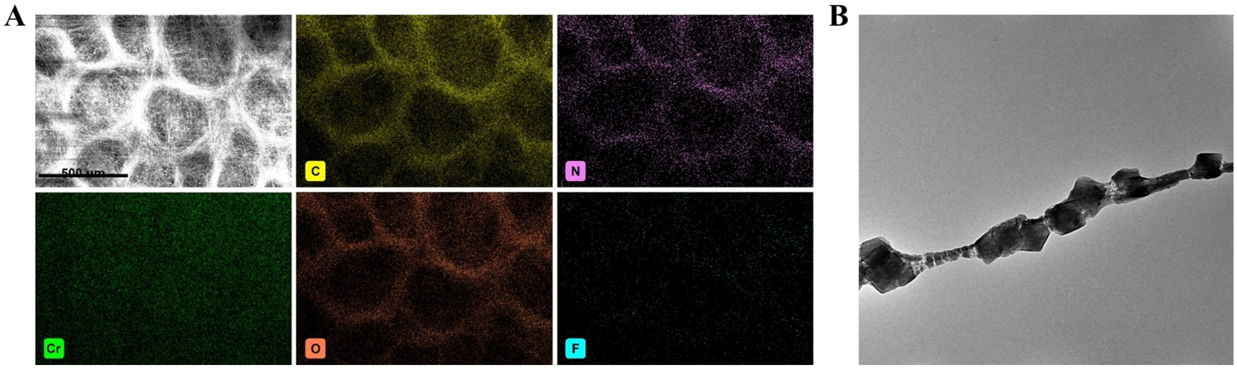

Fig. S3| A) The SEM and element mapping images of honeycomb-like structure after long time outdoor experiment. **B)** TEM image of MIL101(Cr)@PAN.

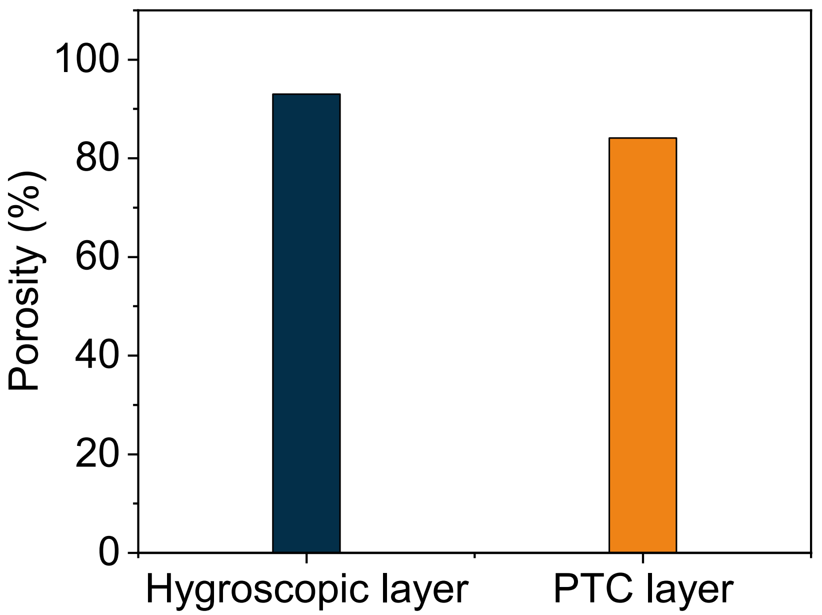

Fig. S4| Porosity of hygroscopic layer and PTC layer.

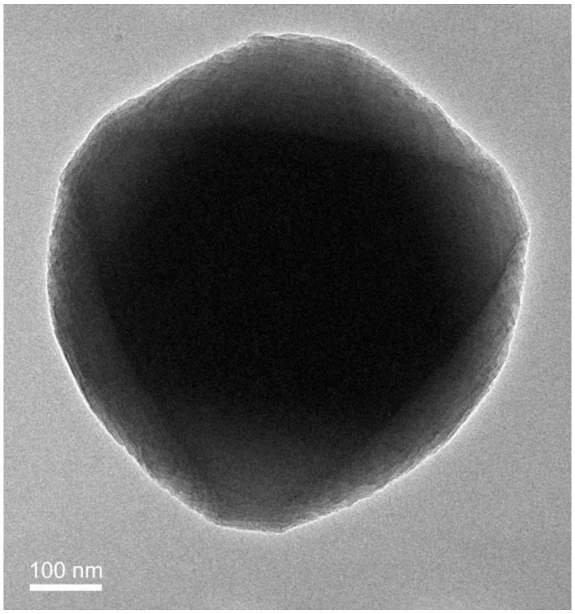

Fig. S5| Transmission electron microscopy (TEM) image of MIL-101(Cr) particle.

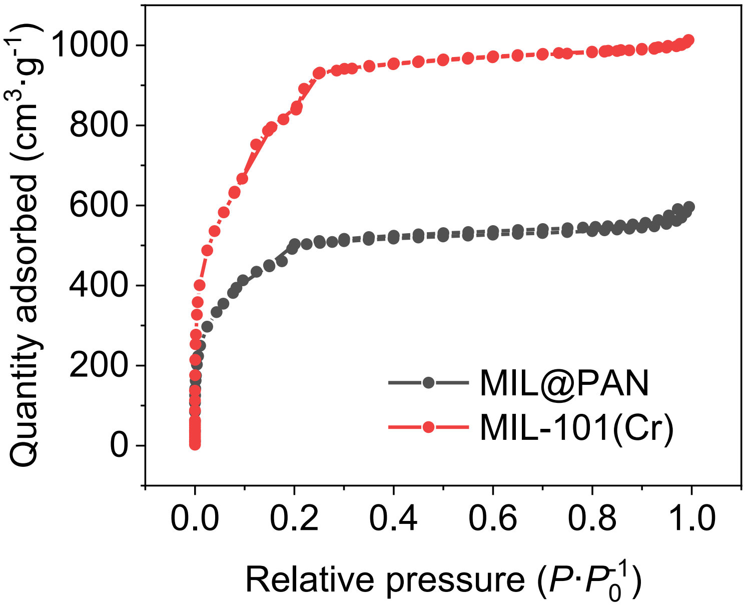

Fig. S6| N_2 adsorption−desorption isotherms curves of MIL-101(Cr) particle and MIL@PAN NFMs

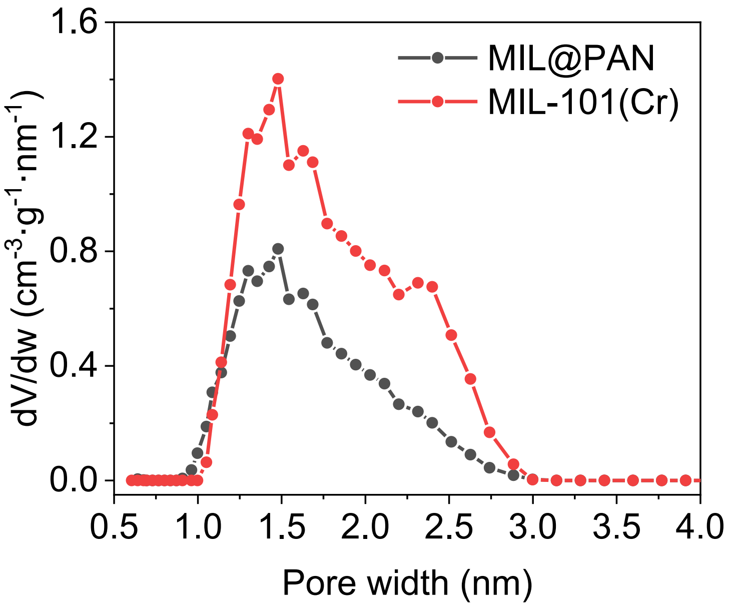

Fig. S7| Pore size distribution curves of MIL-101(Cr) particle and MIL@PAN NFMs

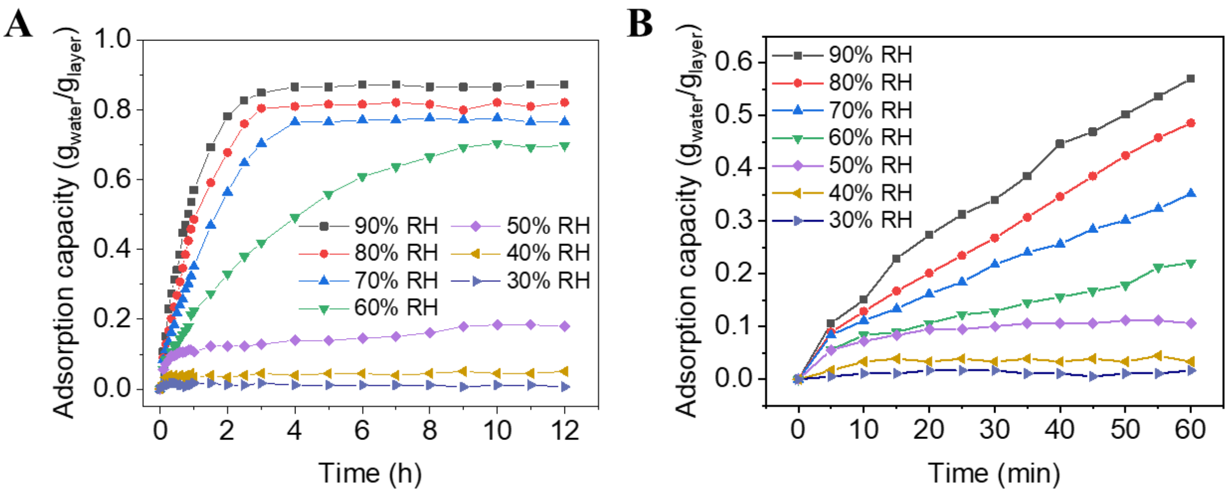

Fig. S8| Moisture adsorption kinetics of hygroscopic layer at 25 °C under various humidities.

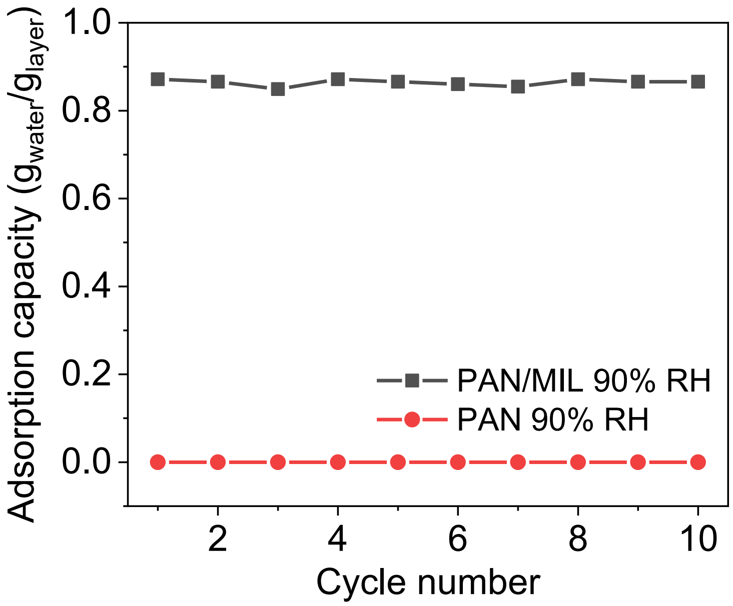

Fig. S9| Cycling stability of moisture adsorption−desorption of MIL@PAN and pure PAN NFMs at 25 °C and 90% RH (desorption at 100 °C).

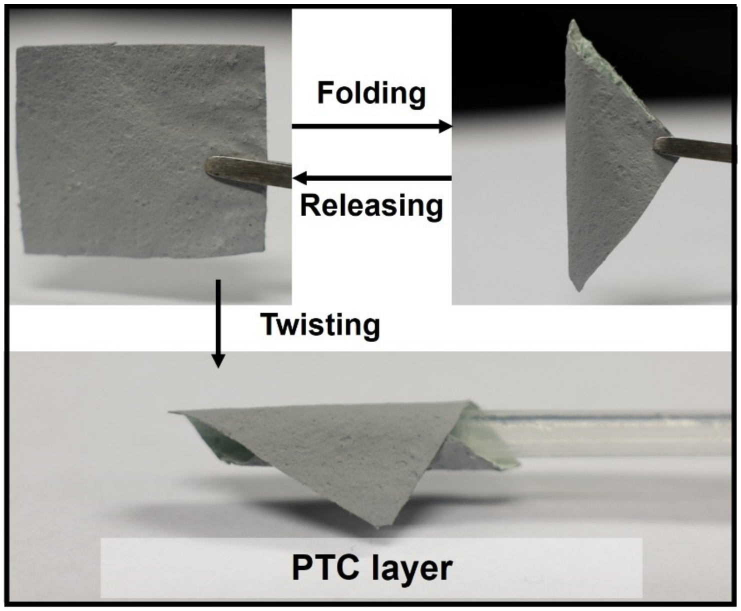

Fig. S10| Photo of flexible photothermal catalytic layer.

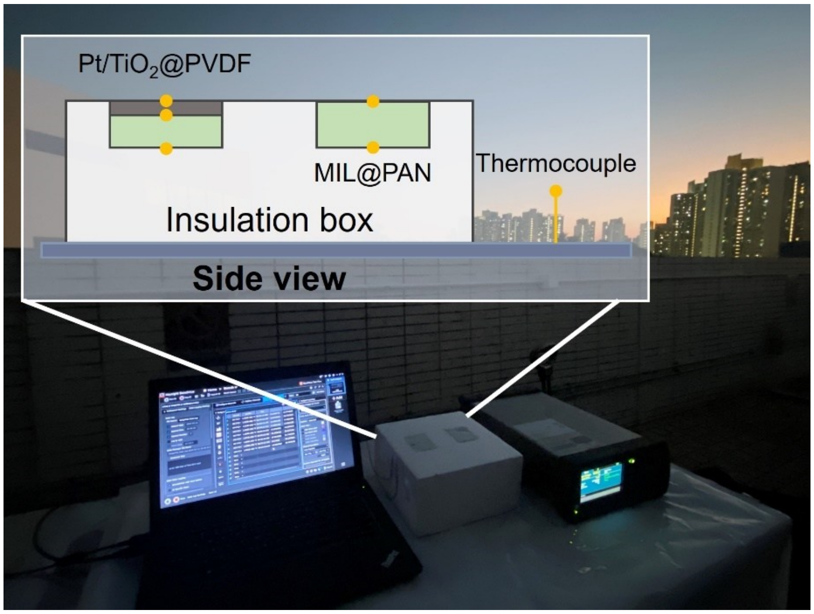

Fig. S11| Photo and schematic of temperature measurement setup for radiative cooling.

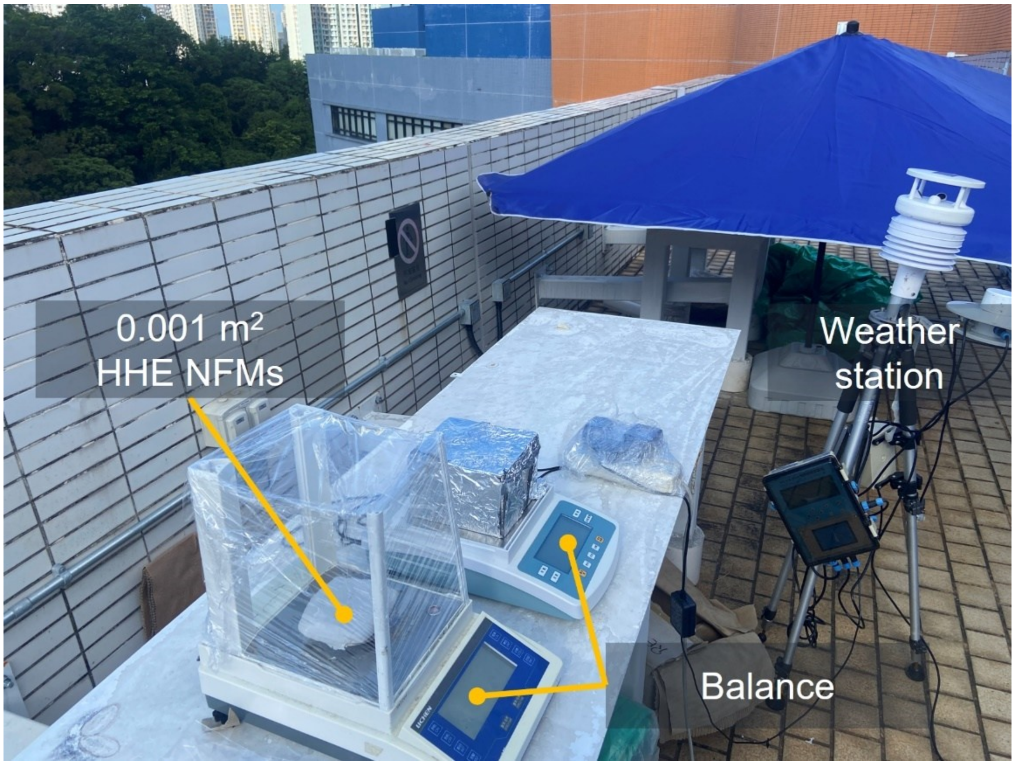

Fig. S12| Photo of the experimental setup used to characterize the moisture adsorption performance of the radiative cooling enhanced system in real-world scenarios.

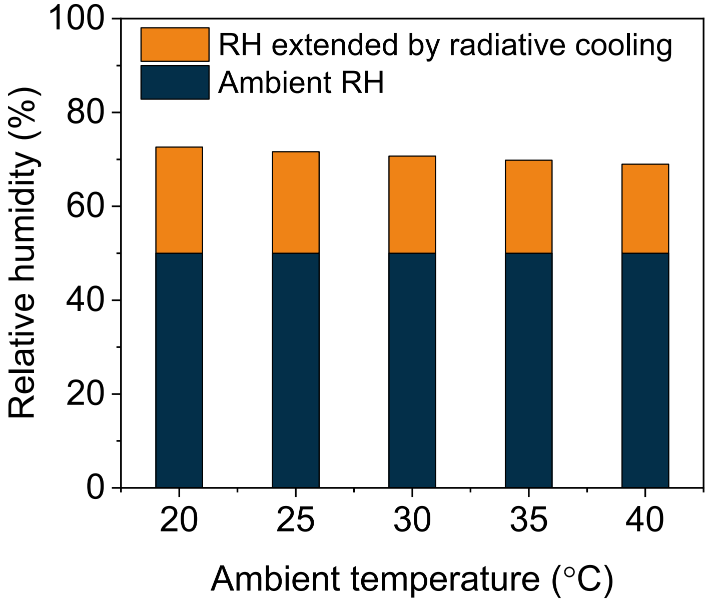

Fig. S13| Effective RH increased by the radiative cooling effect under environmental humidity of 50% RH at various temperature.

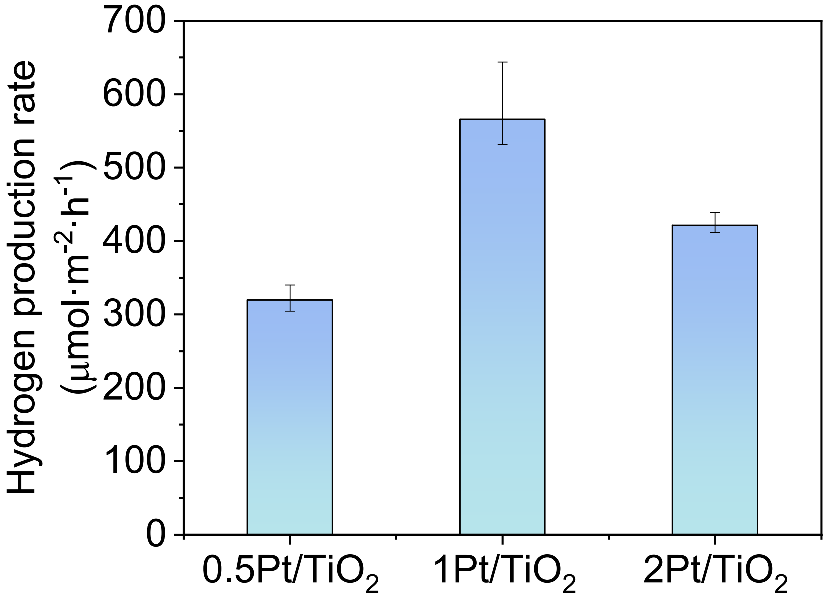

Fig. S14| Optimization of Pt loading mass. Error bars correspond to the standard deviation of three independent measurements.

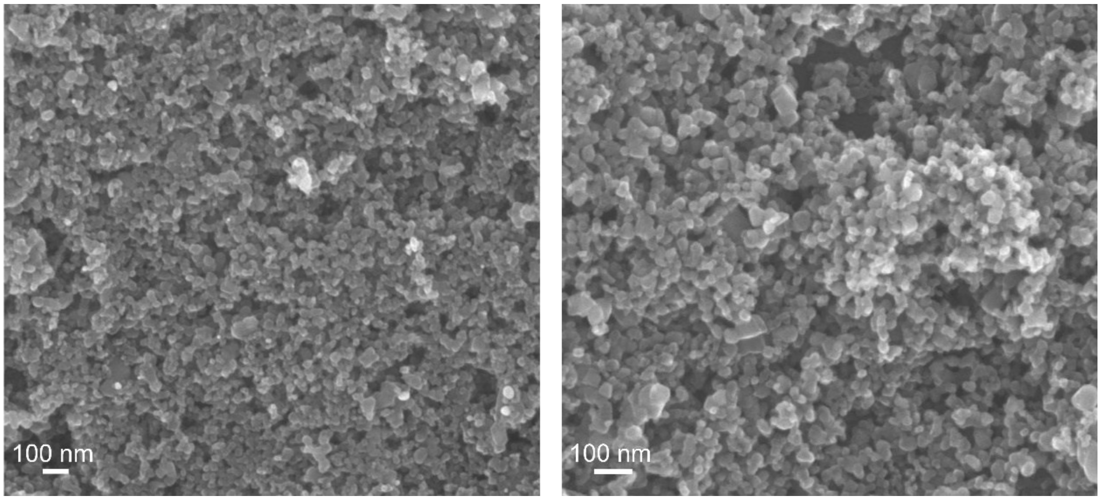

Fig. S15| SEM images of Pt/TiO_2 nanoparticles.

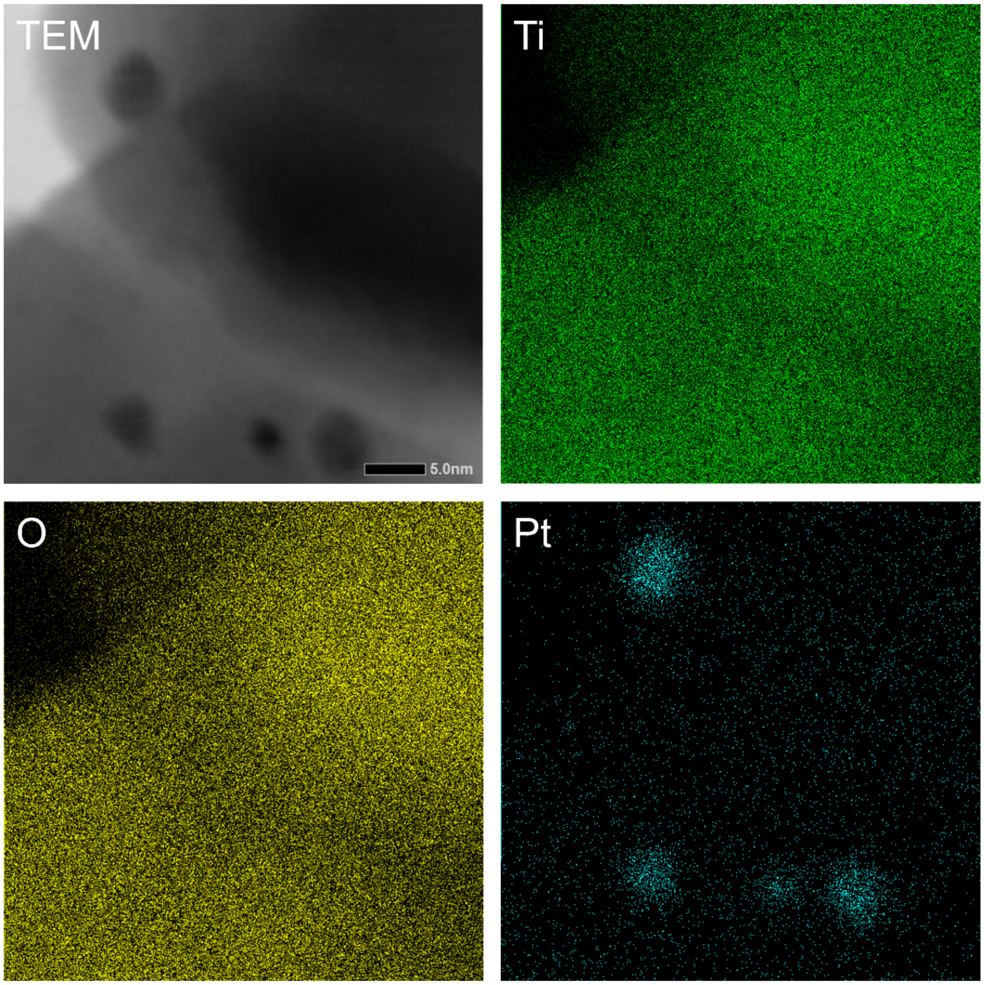

Fig. S16| TEM and element mapping of Pt/TiO_2 nanoparticles.

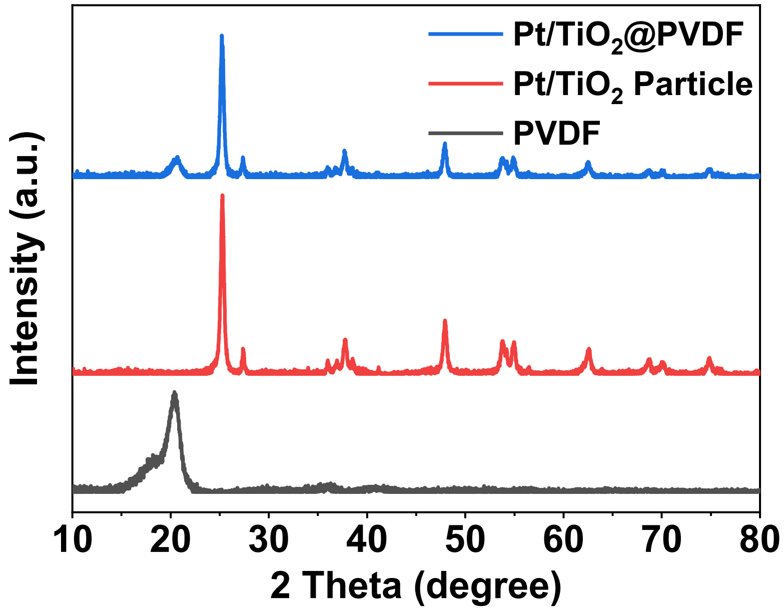

Fig. S17| XRD patterns of Pt/TiO_2@PVDF NFMs, Pt/TiO_2 nanoparticles and PVDF NFMs.

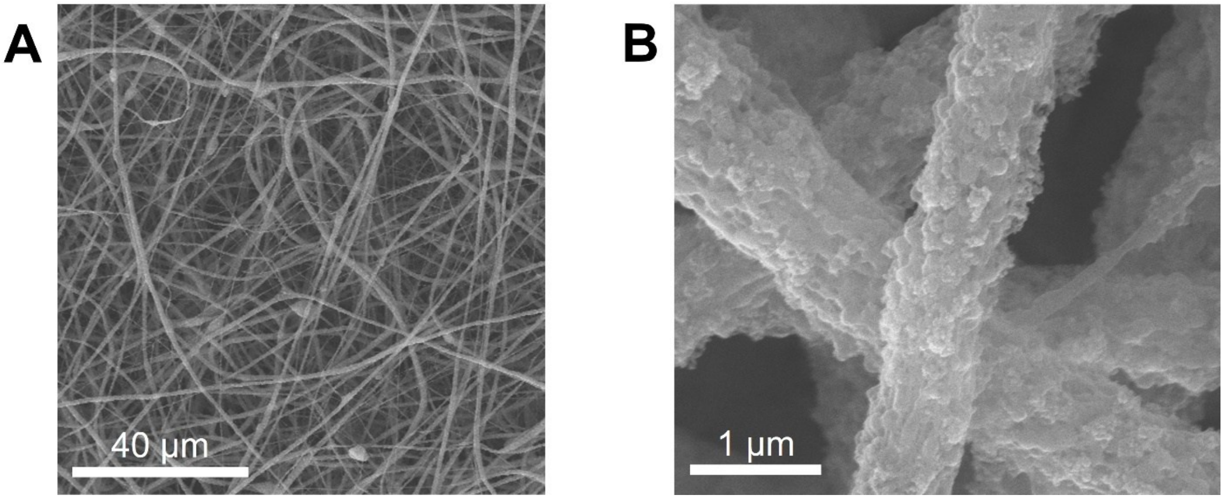

Fig. S18| SEM images of the PTC layer.

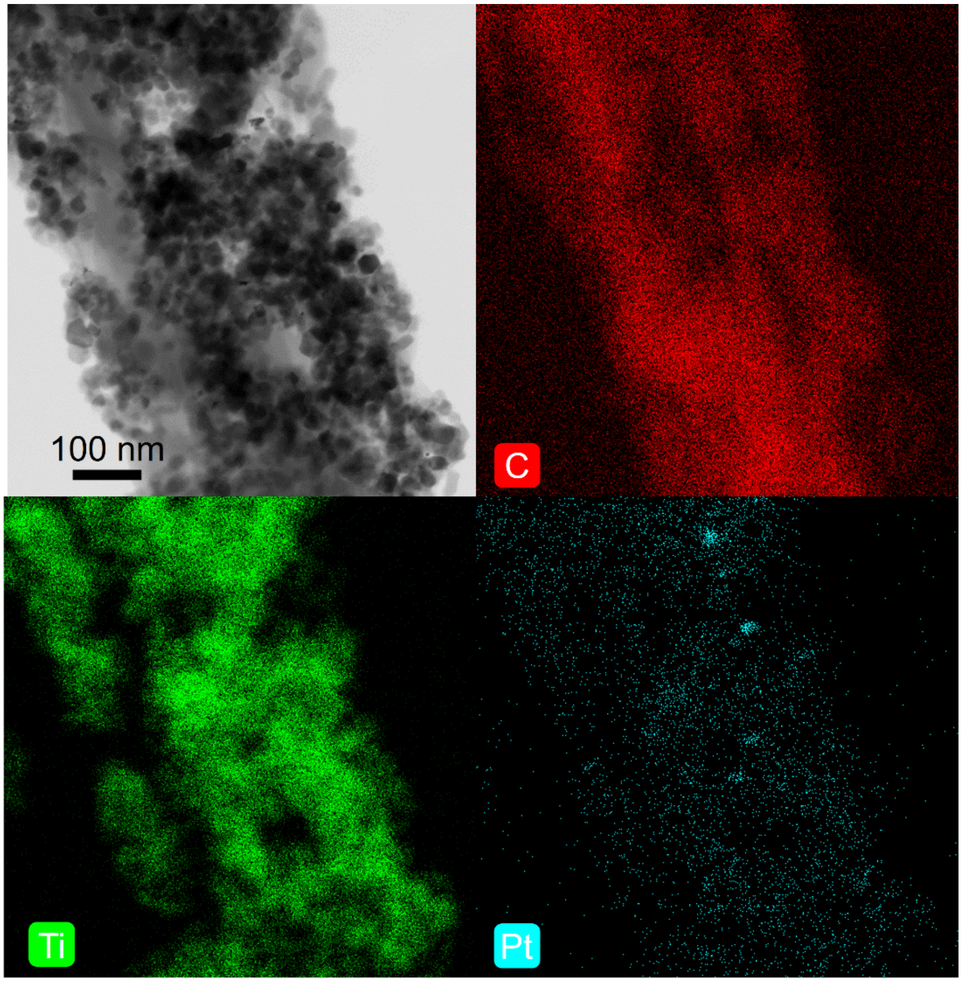

Fig. S19| TEM and element mapping images of the PTC layer.

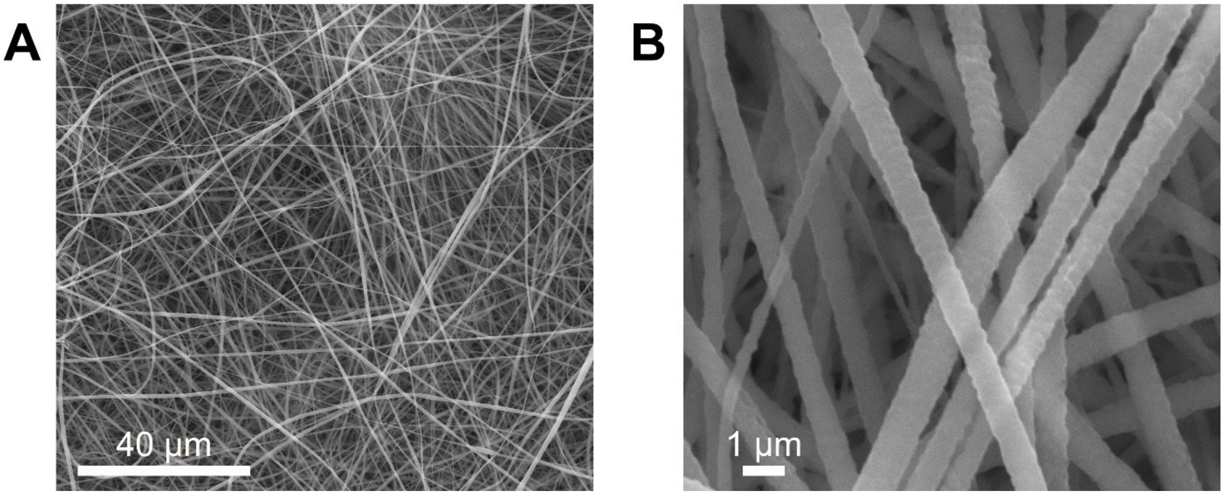

Fig. S20| SEM images of the pure PVDF NFMs.

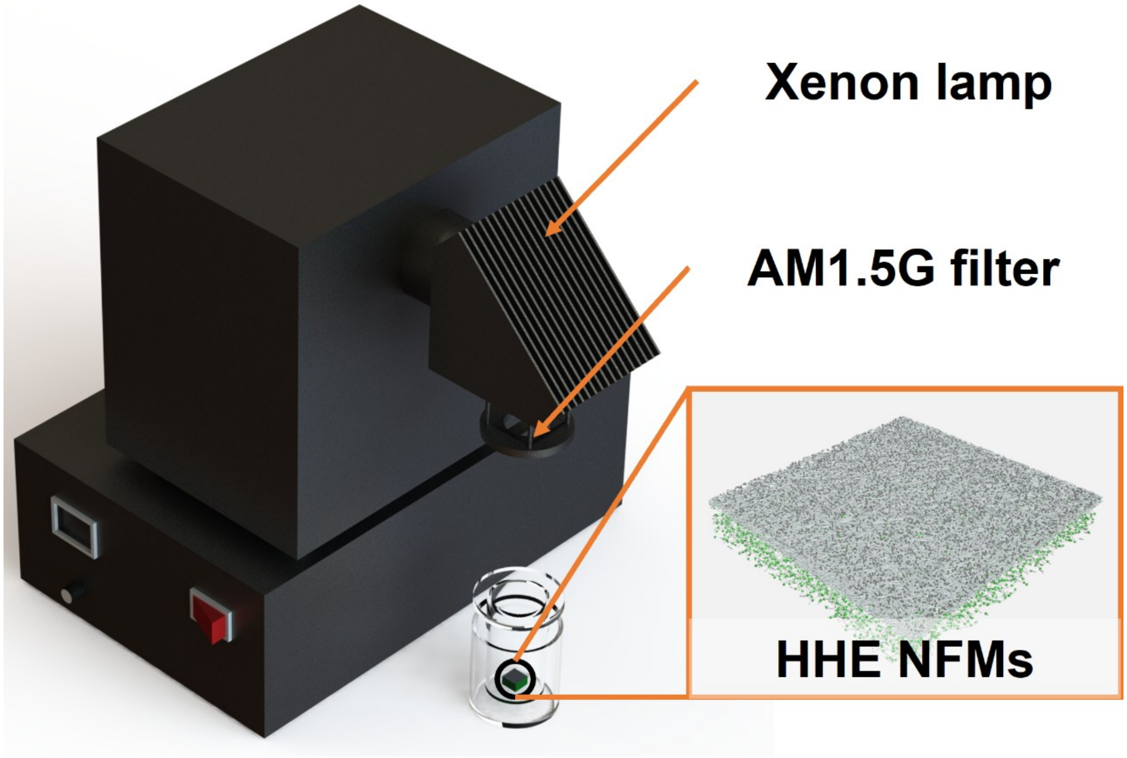

Fig. S21| Concept diagram of H_2 production test

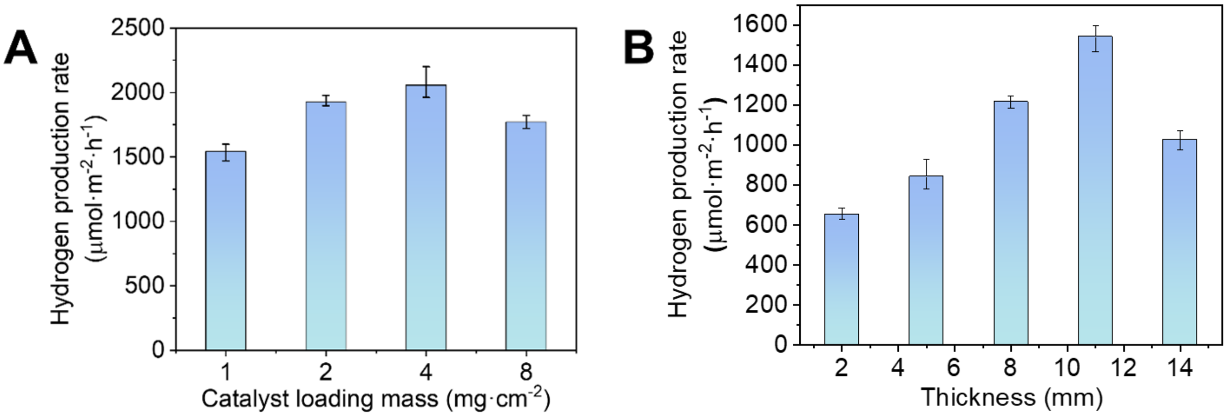

Fig. S22| Optimization of catalyst loading mass and thickness of HHE NFMs for H_2 evolution (Hydrogen production rate during the first hour) (The units $\mu mol \cdot m^{-2} \cdot h^{-1}$ represent μmol per square meter HHE NFMs per hour). Error bars correspond to the standard deviation of three independent measurements.

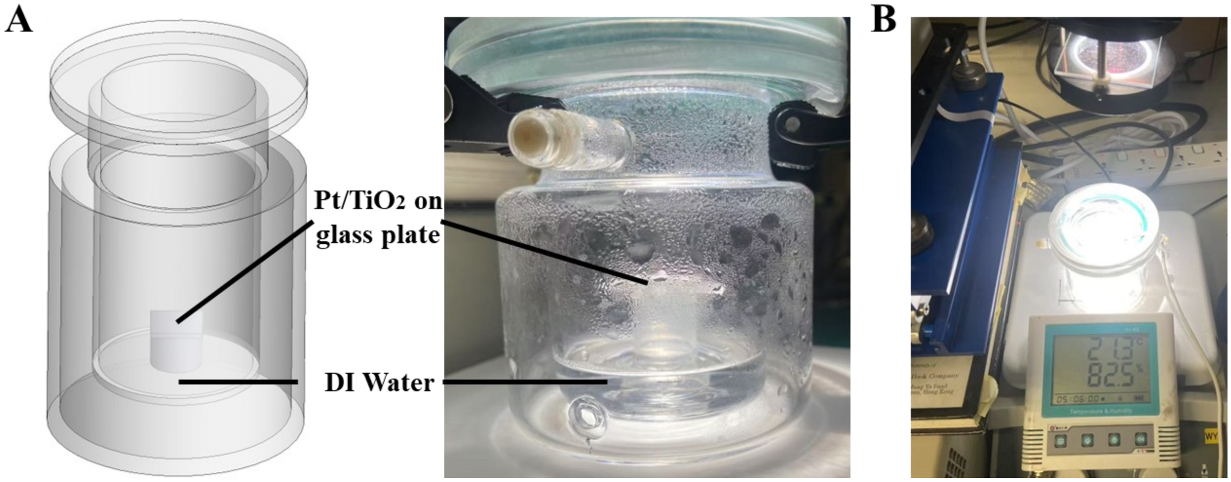

Fig. S23| A) Schematic and actual setup of biphasic system with vapor feeding. **B)** The record of RH in the biphasic photoreactor.

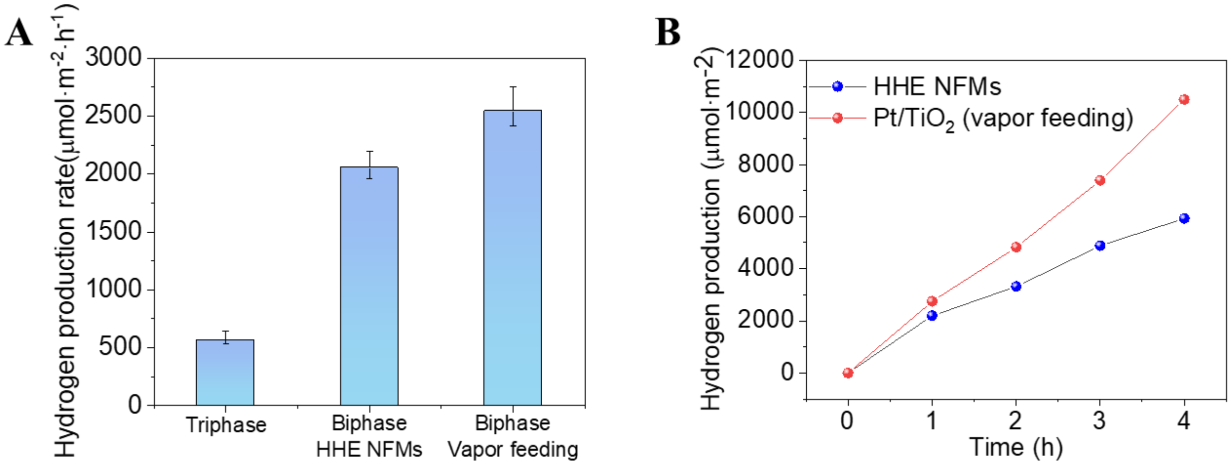

Fig. S24| A) H_2 production rate comparison of triphasic and biphasic reaction system in the first hour. **B)** H_2 production comparison of HHE NFMs biphasic system and biphasic system with vapor feeding. Error bars correspond to the standard deviation of three independent measurements.

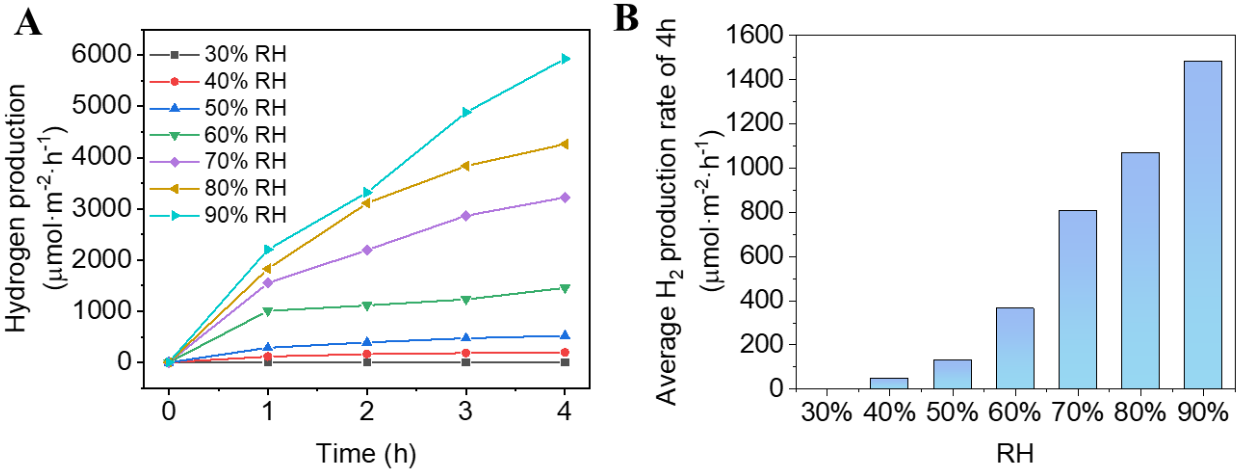

Fig. S25| H_2 production rate of HHE NFMs (0.0004 m^2) under indoor simulated sunlight. d) Average H_2 production rate of 4h sunlight illumination.

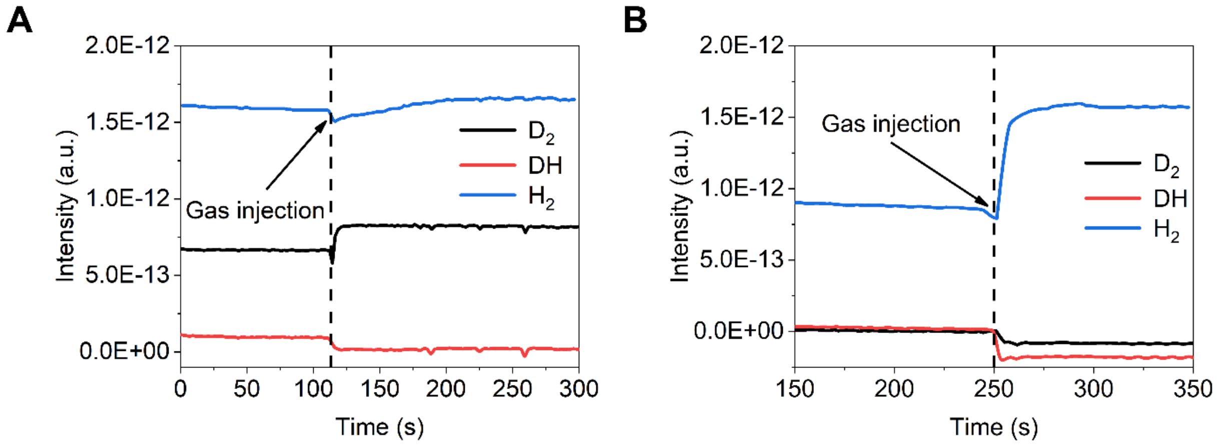

Fig. S26| Mass spectrometer signals of gas generated from HHE NFMs after illumination by using A) D_2O, **B)** H_2O.

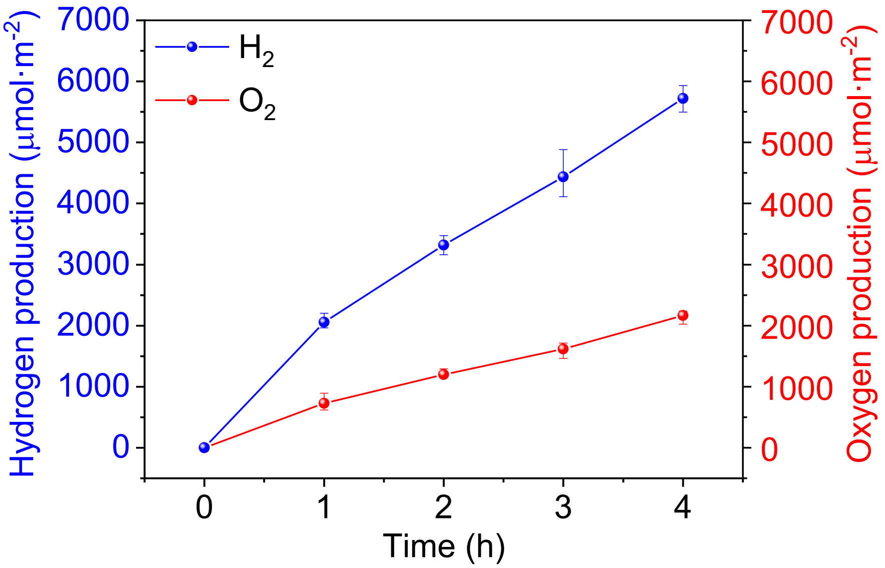

Fig. S27| Time dependent gas production from HHE NFMs under AM1.5G simulated solar light. Error bars correspond to the standard deviation of three independent measurements.

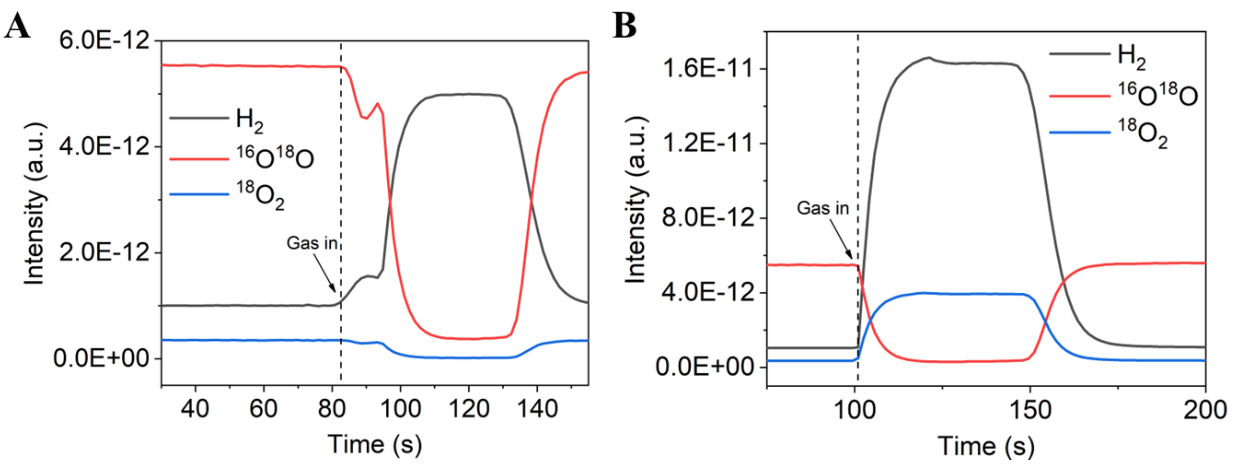

Fig. S28| Mass spectrometer signals of gas generated from HHE NFMs after illumination by using A) $H_2{}^{18}O$**, B)** $H_2{}^{16}O$

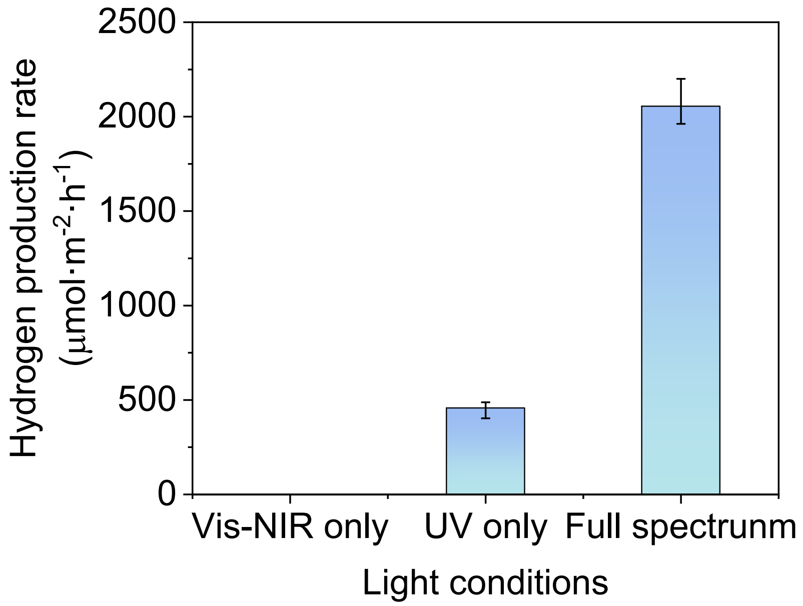

Fig. S29| H_2 production rate under different light conditions during the first hour. Error bars correspond to the standard deviation of three independent measurements.

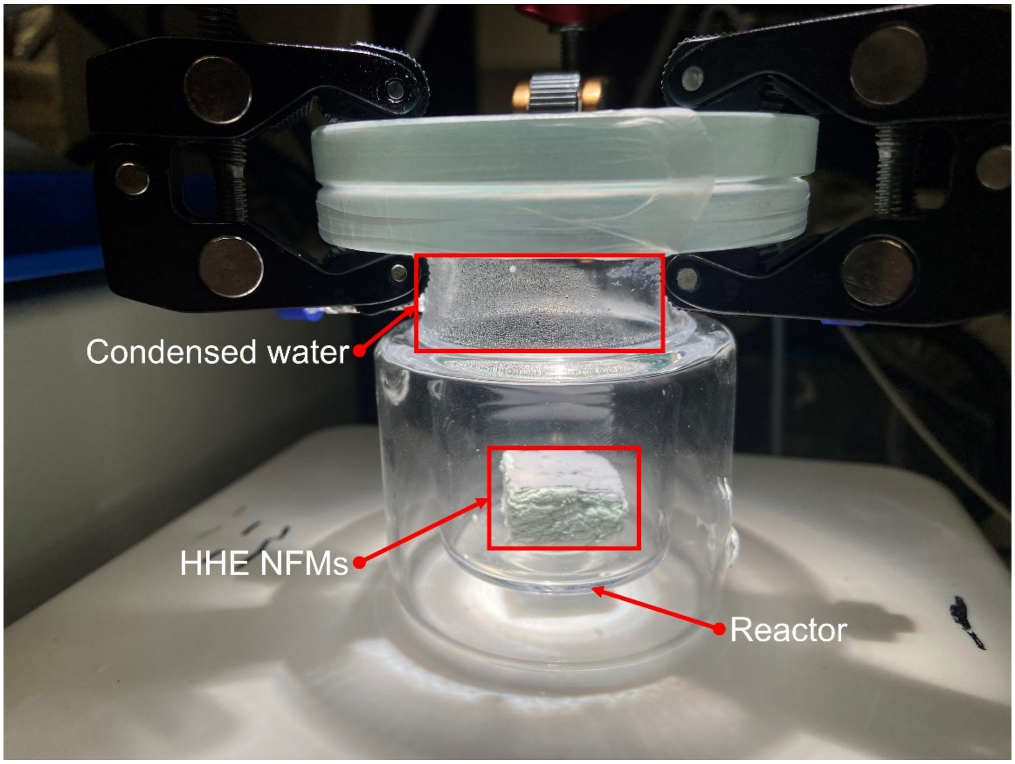

Fig. S30| Photo of H_2 production test under Vis-NIR light condition with condensed water on the wall of reactor.

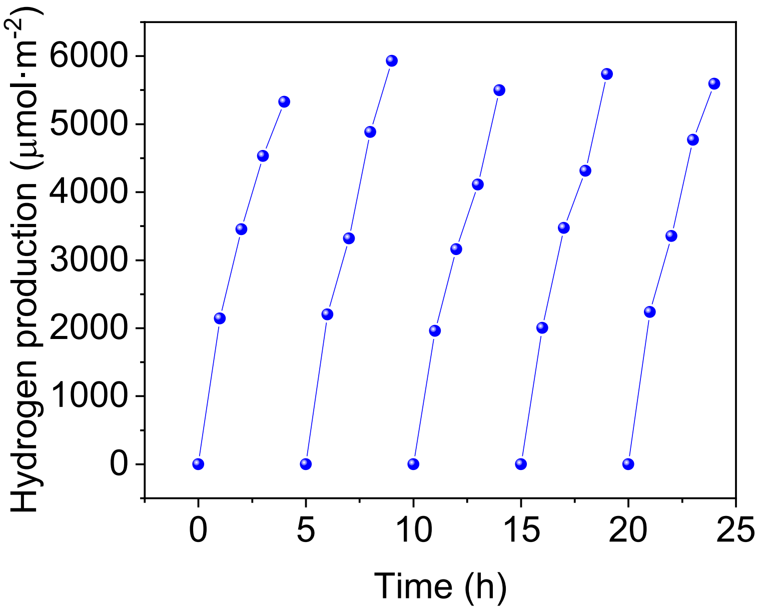

Fig. S31| H_2 production cycling stability of HHE NFMs.

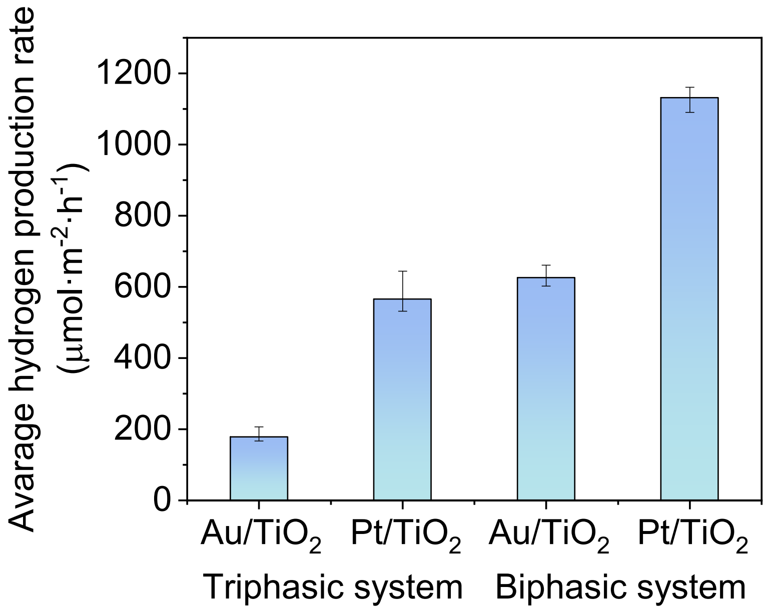

Fig. S32| H_2 production rate over 6h of Au/TiO_2 and Pt/TiO_2 in triphasic and biphasic system.

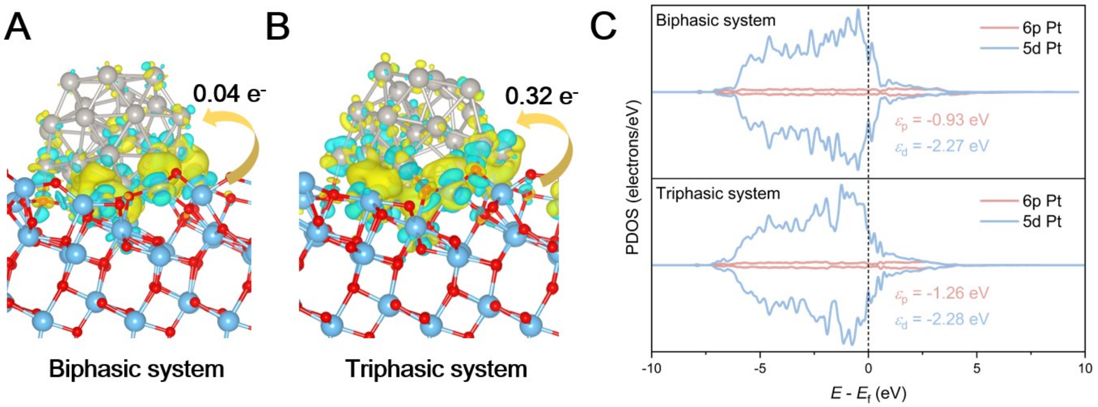

Fig. S33| The charge density difference and the corresponding electron transfer analyzed by Bader charge for A) biphasic system and **B)** triphasic system (The silver, blue and red balls indicate Pt, Ti and O, respectively. The yellow and blue colors represent charge accumulation and depletion. The isosurface values are set at ±5.0×10-3 e/Å3). **C)** PDOS for Pt/TiO_2 of biphasic system and triphasic.

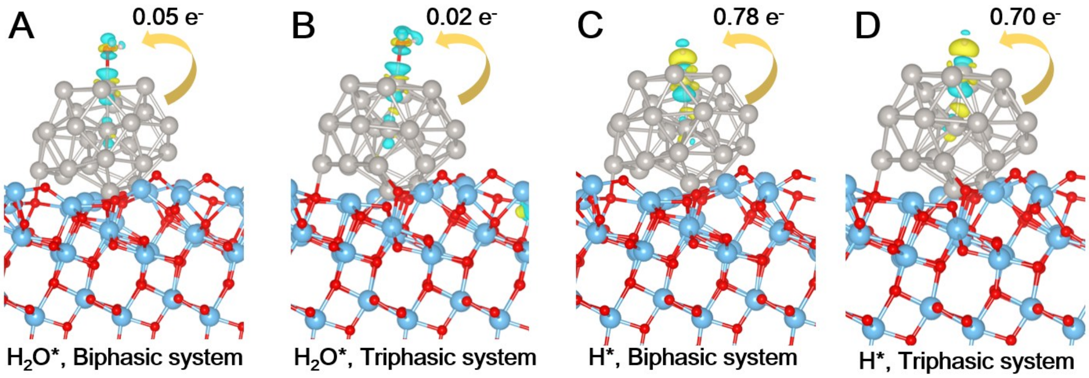

Fig. S34| The charge density difference and the corresponding electron transfer analyzed by Bader charge for A) adsorbed H_2O* of biphasic system and **B)** adsorbed H_2O* of triphasic system, **C)** adsorbed H* of triphasic system and **D)** adsorbed H* of triphasic system (The silver, blue, red and white balls indicate Pt, Ti, O and H, respectively. The yellow and blue colors represent charge accumulation and depletion. The isosurface values are set at ±5.0×10-3 e/Å3).

The charge density difference (CDD) and projected density of states (PDOS) results show that Pt cluster of biphasic system accept less electron from TiO_2 (101) substrate, leading to upshift of p orbital and d orbital center compared to that of triphasic system (Fig. S33). As a result, the adsorption strength, and electron transfer of water molecule and hydrogen atom of biphasic system both are larger than those of triphasic system under the same temperature (298 K) (Fig. S34).

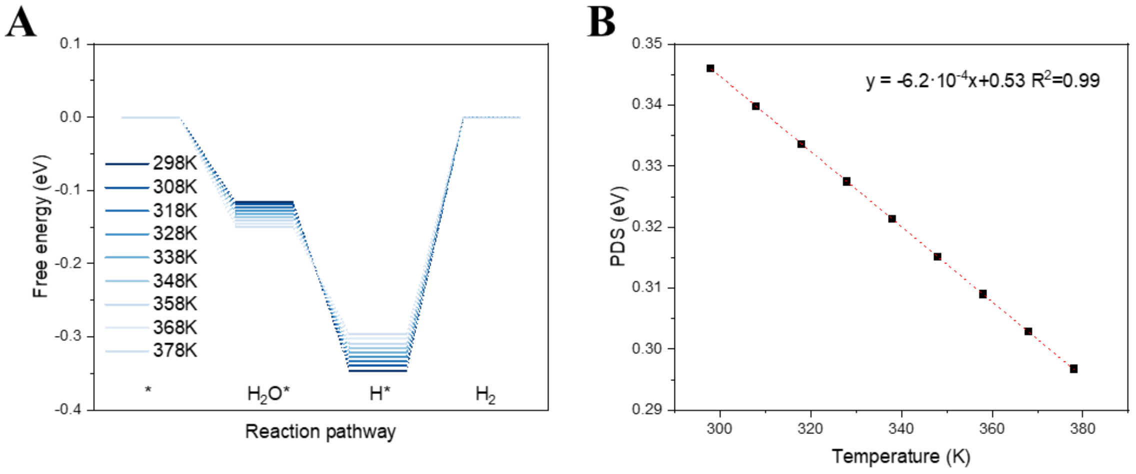

Fig. S35| A) Calculated Gibbs energy of a photocatalytic reaction in the biphasic system over the Pt/TiO_2 surface at various temperature**. B)** Reaction energy barriers of potential determining step (PDS) for the biphasic system at various temperature.

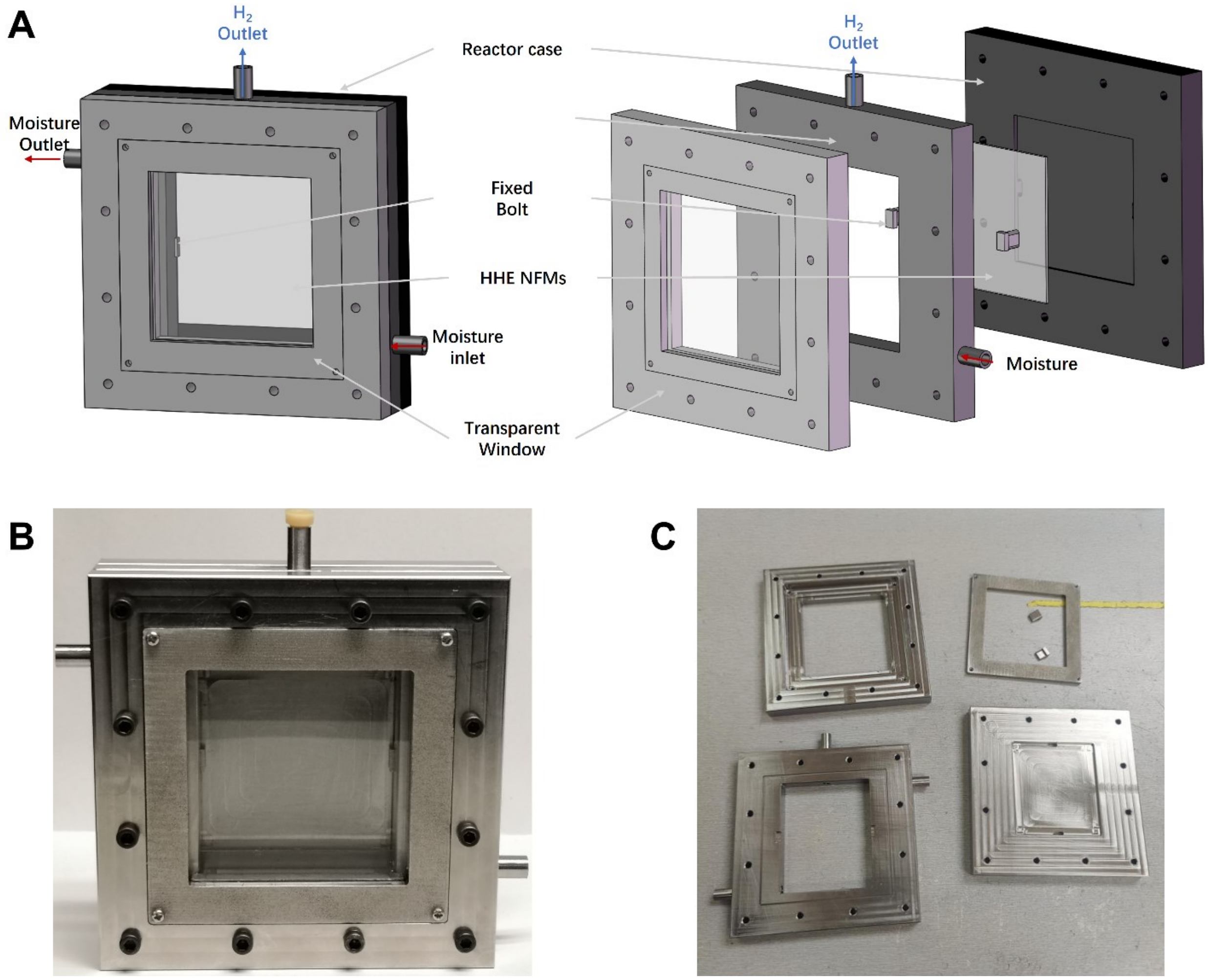

Fig. S36| The scaled-up HHE reactor for 0.002 m^2 HHE NFMs. A) 3D model of the reactor**, B) and C)** Photo of the reactor**.**

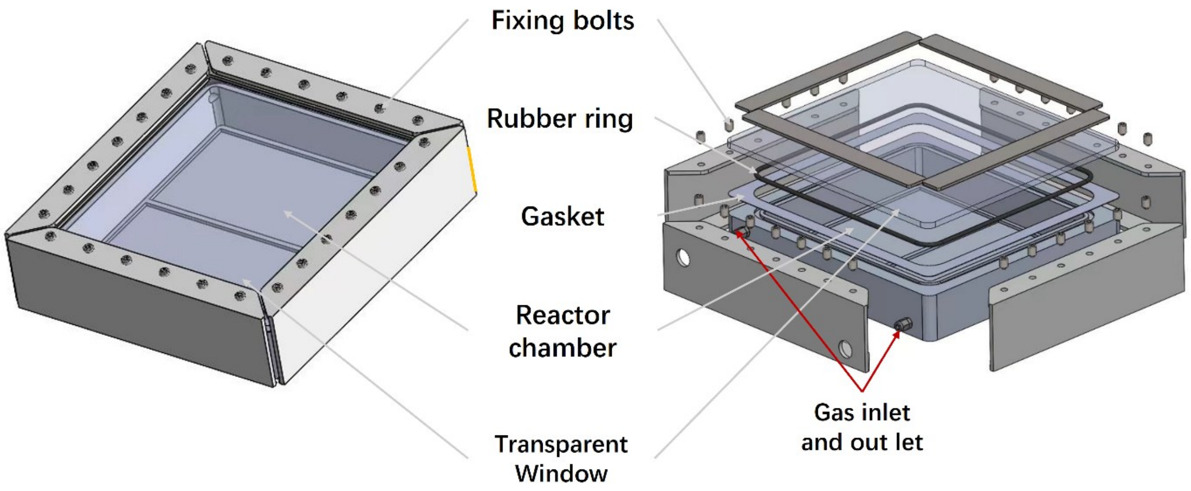

Fig. S37| The scaled-up HHE reactor for 4 pieces of 0.01 m^2 HHE NFMs arranged in an array within the reactors.

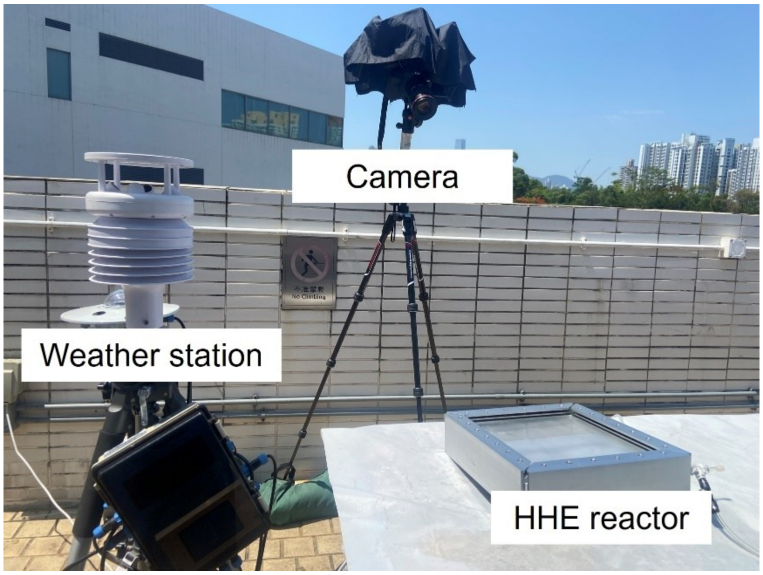

Fig. S38| The scaled-up HHE reactor (Fig. S27) was exposed to natural light to produce H_2.

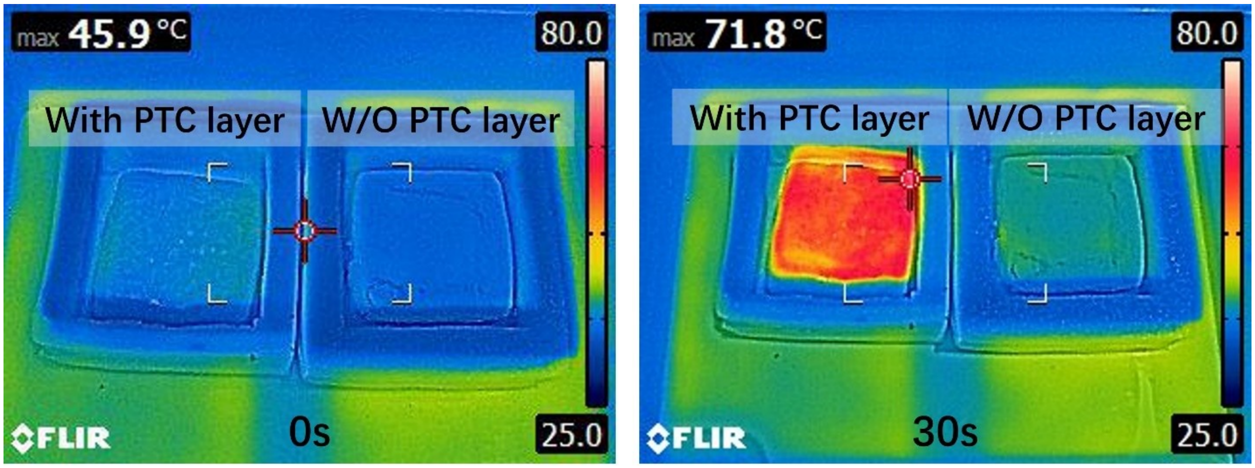

Fig. S39| IR thermal images revealing the top surface temperature of HHE NFMs (left: initial state without sunlight, right: exposed to sunlight).

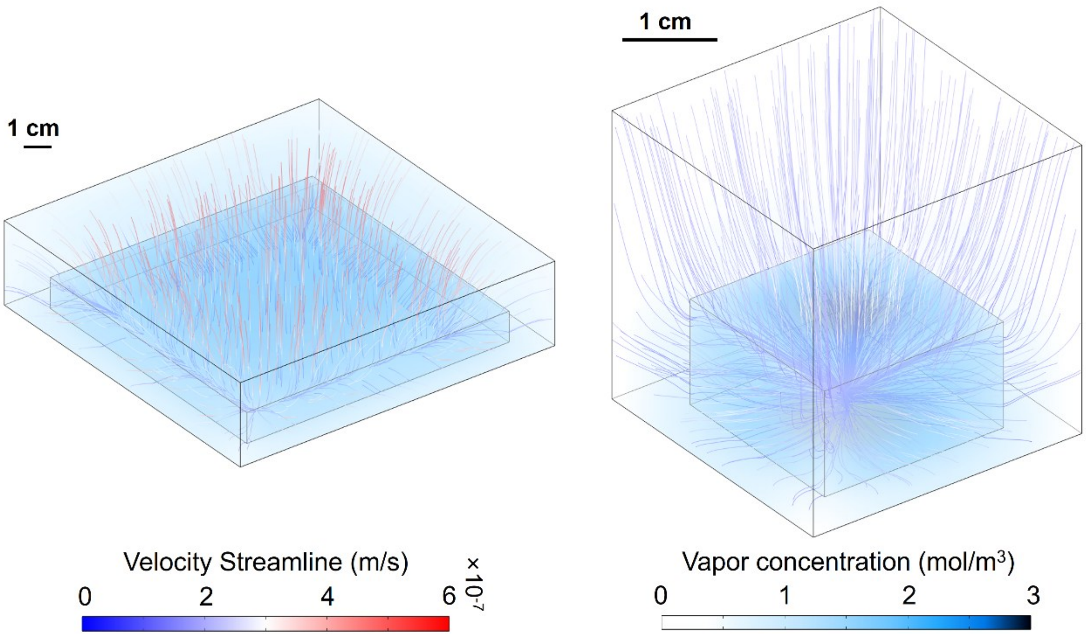

Fig. S40| The velocity distribution of water vapor streamlines and concentration cloud maps of HHE NFMs (3D simulation results)

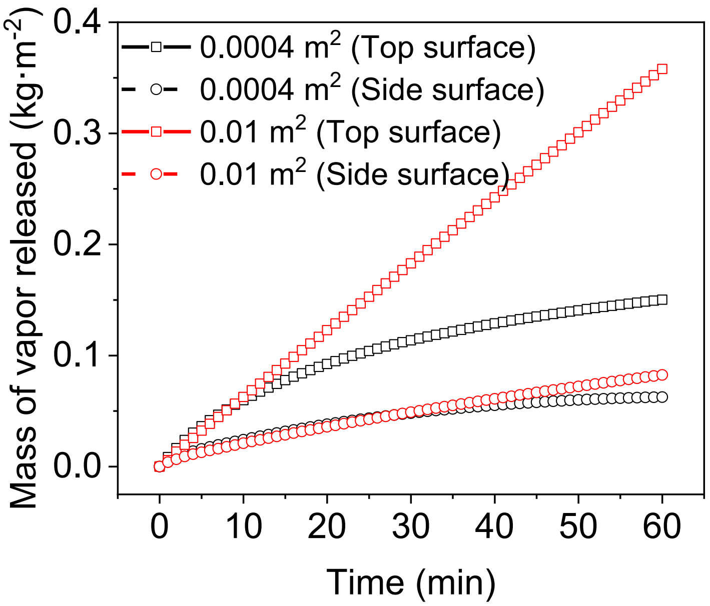

Fig. S41| Comparison of water vapor release over time from the top and side surfaces of HHE NFMs with different sizes obtained through 3D numerical calculations.

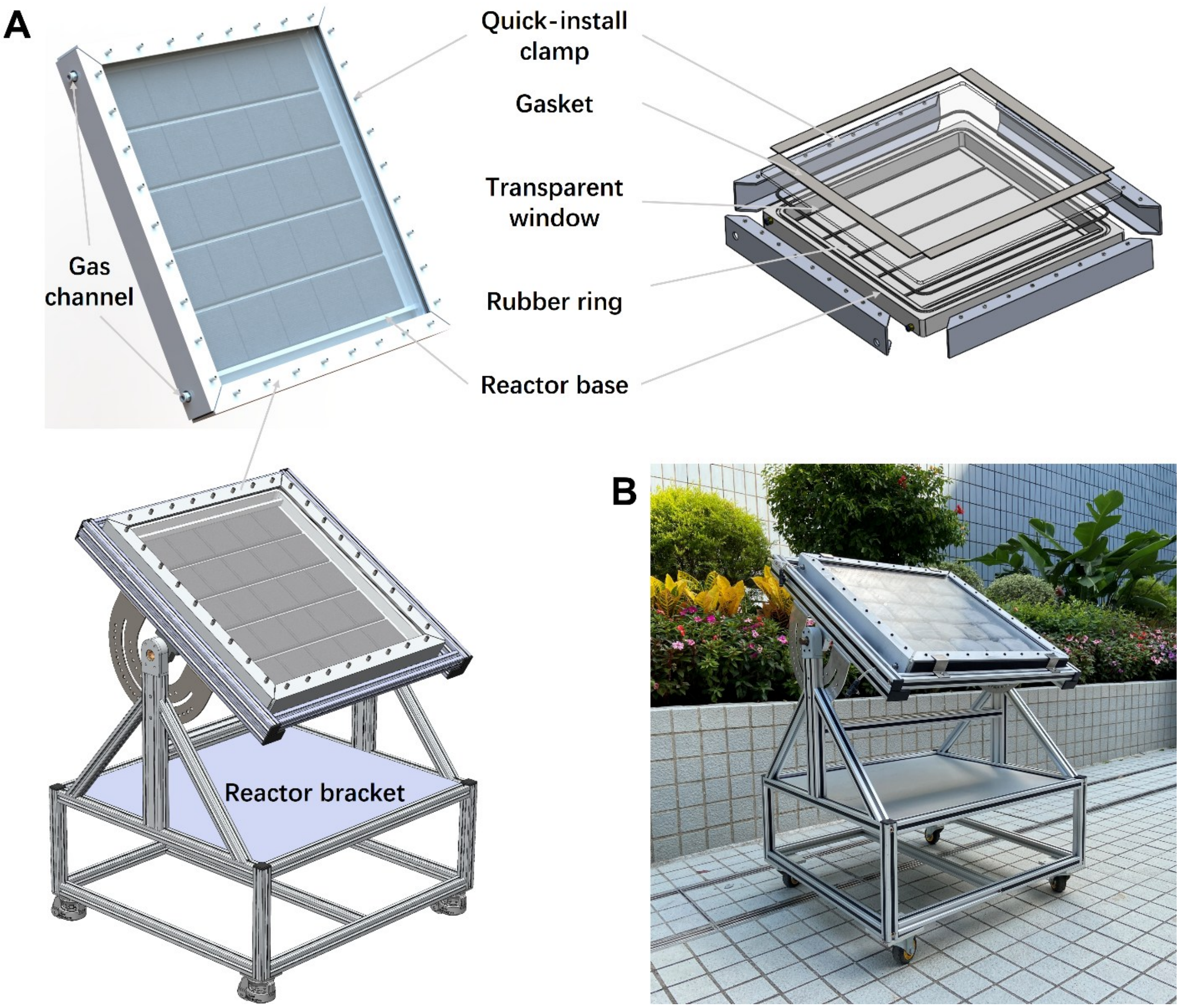

Fig. S42| Large-scale HHE reactor with 25 pieces of 0.01 m^2 HHE NFMs arranged in an array within the reactors. A) 3D model of large-scale HHE system. **B)** Photo of the large-scale HHE system.

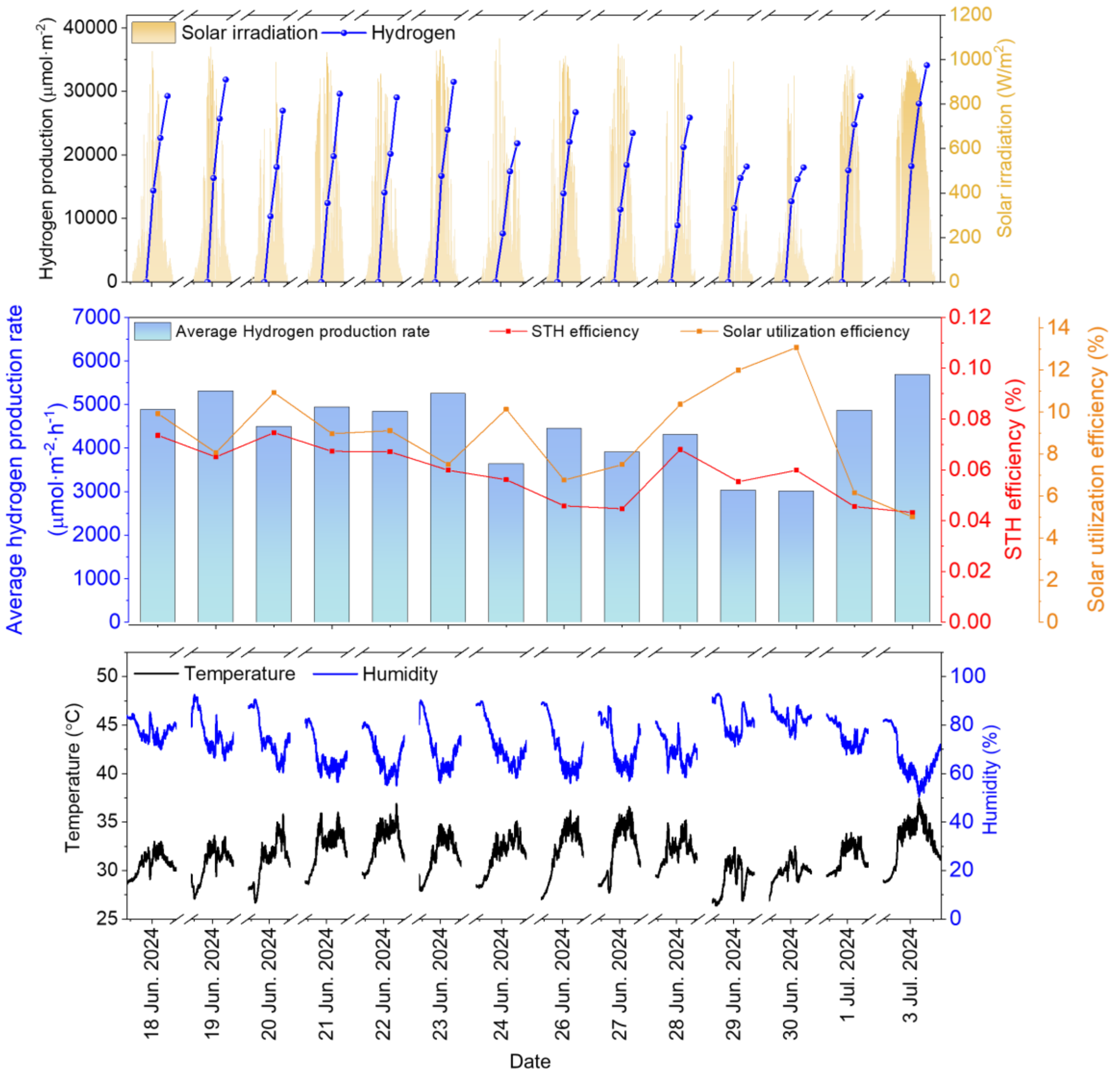

Fig. S43| Operational status of the large-scale HHE system over a 14-day period. H_2 production, solar radiation intensity, H_2 production rate and STH efficiency and Solar utilization efficiency at corresponding humidity and temperature during the 14-day period.

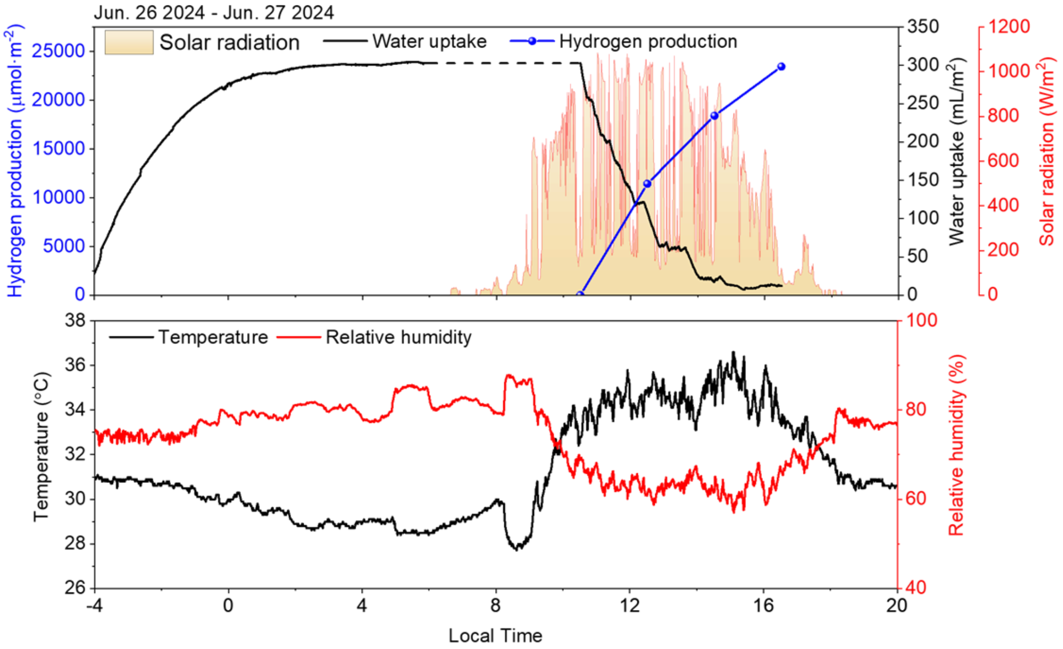

Fig. S44| 24h-continuous moisture adsorption and desorption test and H_2 production test after long time outdoor experiment, with the humidity, temperature, and solar radiation intensity.

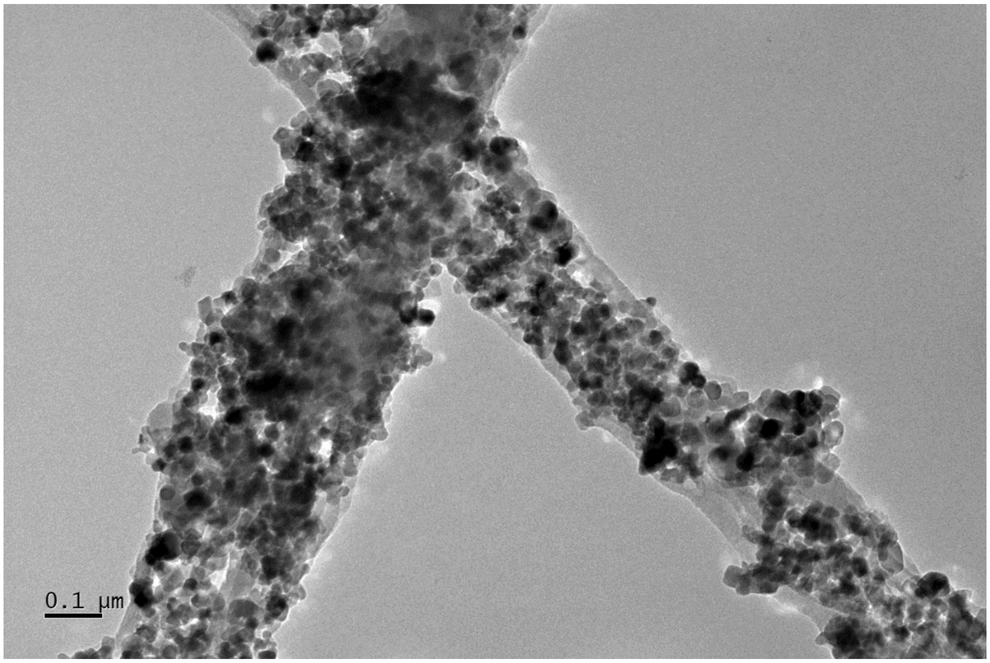

Fig. S45| The TEM photo of PTC layer after long time outdoor experiment.

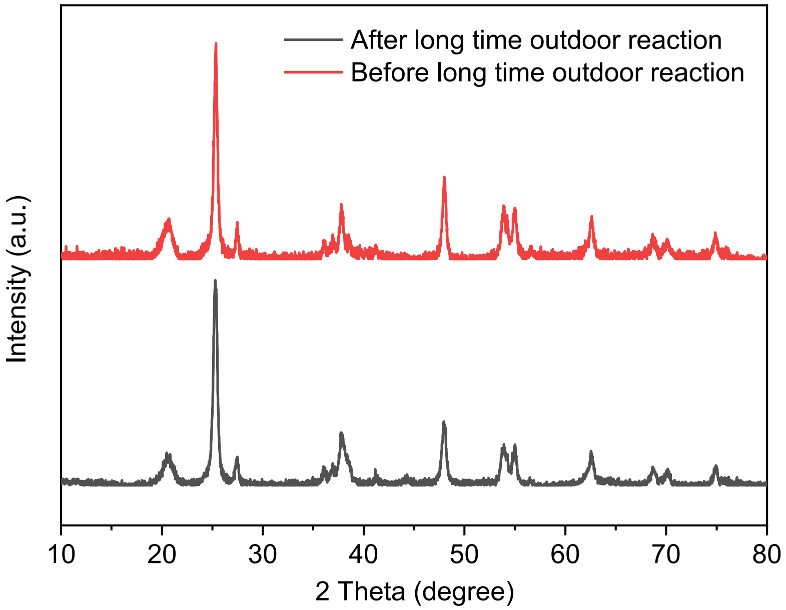

Fig. S46| The XRD test result of PTC layer before and after long time outdoor experiment.

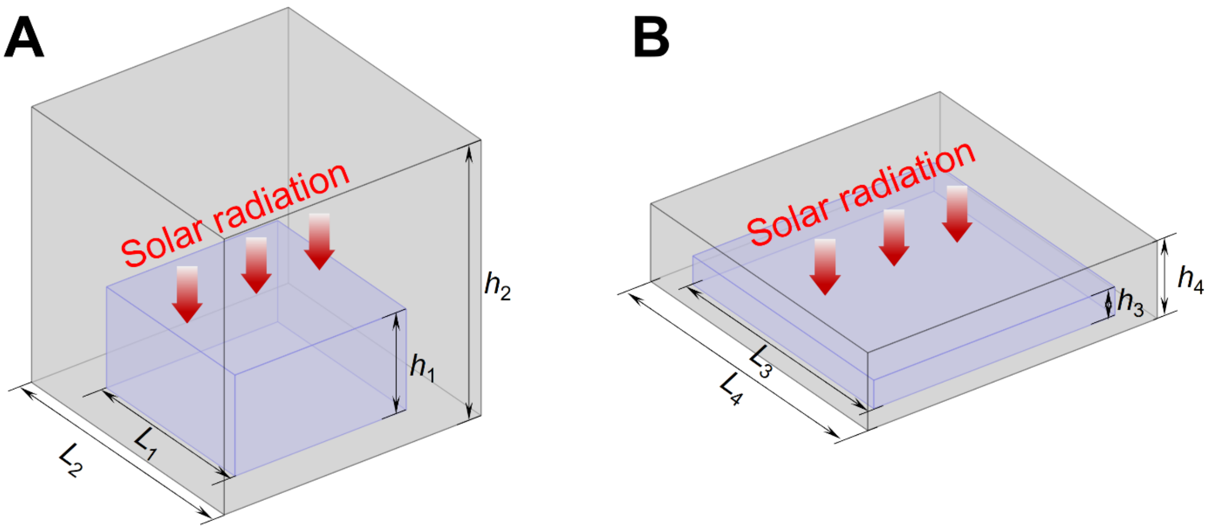

Fig. S47| Geometric parameters of 3D model construction. (**A**) 3D model of 4 cm^2 HHE NFMs for water desorption process. (**B**) 3D model of 100 cm^2 HHE NFMs for water desorption process. The blue areas are HHE NFMs with different size, which consists of porous layers. The one sun illumination is directed at the top of HHE NFMs. The gray area represents the vapor diffusion layer. The generated vapor can pass through the vapor diffusion layer and exits beyond the boundary. L_1= 2 cm, L_2=3 cm, h_1=1.1 cm, h_2=3 cm, L_3= 10 cm, L_4=12 cm, h_3=1.1 cm, h_4=3 cm.

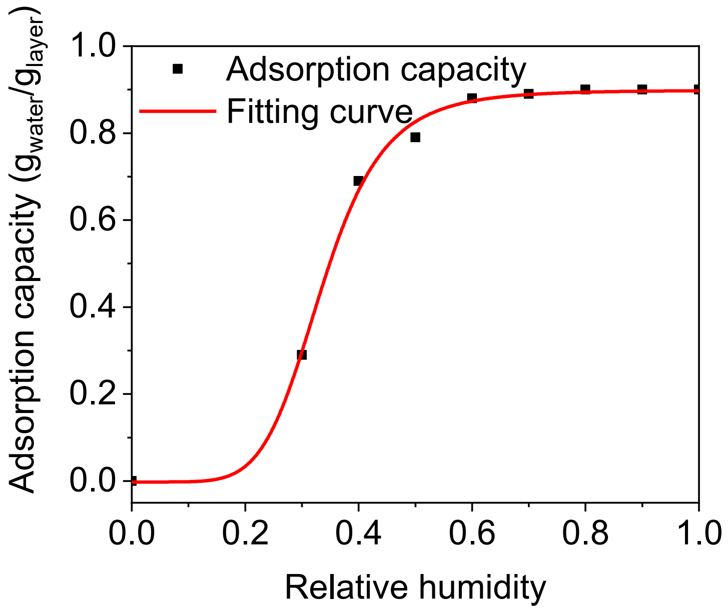

Fig. S48| The relationship between RH and the water adsorption capacity.

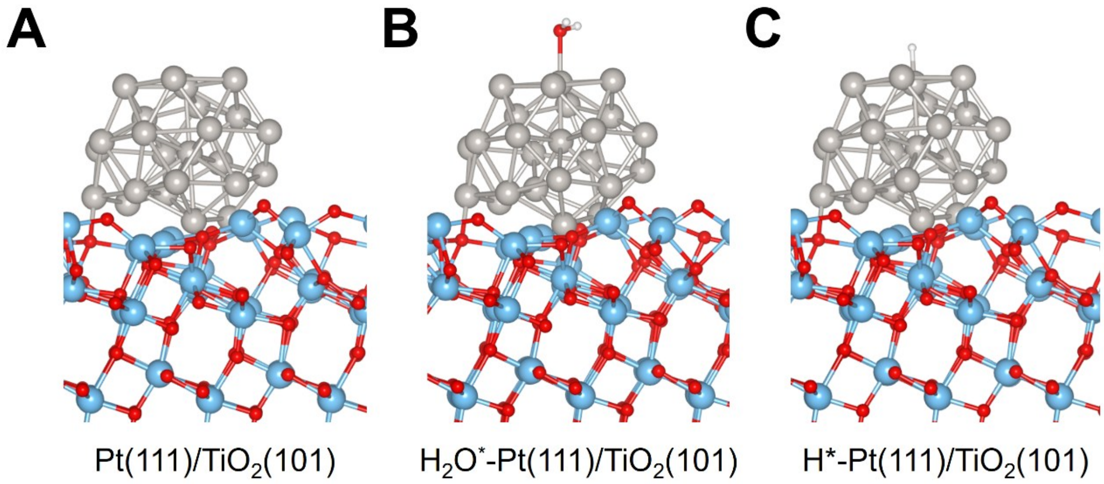

Fig. S49| The optimization structures of (A) Pt(111)/TiO_2(101), **(B)(C)** H_2O and H adsorbed on Pt;

Note S1 Specific definition of different weather conditions

A) Sunny conditions (with average solar intensity is around 786.0 $W \cdot m^{-2}$): With minimal cloud coverage throughout the testing period.

B) Partially cloudy conditions (with average solar intensity around 716.5 $W \cdot m^{-2}$): Featuring intermittent cloud passage causing temporary shading of the reactor.

C) Overcast conditions (with average solar intensity around 397.6 $W \cdot m^{-2}$): With persistent cloud coverage frequently obstructing solar irradiation.

The comprehensive time-resolved data for solar irradiance and temperature during each weather condition have been provided in manuscript Fig. 4b.

Note S2 The analyze of the enhancement of hydrogen production rate form thermodynamic perspective:

A comprehensive energy analysis of the photothermal catalytic (PTC) layer surface reveals that under outdoor conditions, the upper surface absorbs significantly more solar energy, leading to elevated surface temperatures. This thermal effect thermodynamically favors the hydrogen evolution reaction. The specific mechanisms are elaborated as follows:

(1) Discrepancies in spectral intensity distribution between indoor simulated sunlight and outdoor natural light

First, we provide detailed specifications of the simulated solar light source used in indoor experiments (Fig. S50). The Xenon lamp system (Newport Model 67005 with Power Supply 69911) equipped with an AM1.5G filter was employed. Figure S50 presents the spectral irradiance and our measured spectral distribution when using the AM1.5G filter. Notably, comparative analysis reveals that, in the ultraviolet range and visible range (625-800 nm), the filtered xenon lamp exhibits higher irradiance than natural sunlight. However, in the 450-625 nm of visible light, filtered xenon lamp exhibits lower irradiance. While these spectral mismatches were shown in the figure, these differences alone cannot fully explain the six times increase in her performance observed under outdoor conditions.

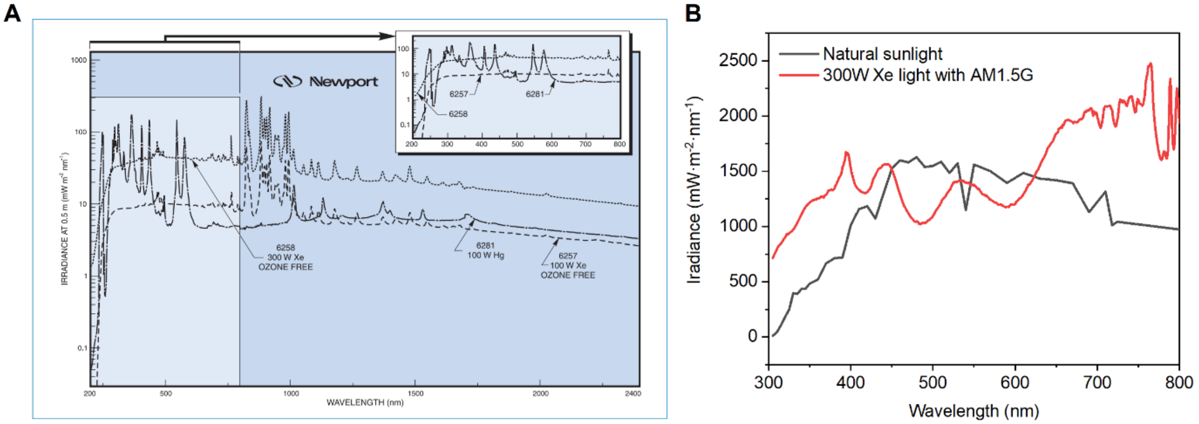

Fig. S50| A) The spectral irradiance of various Arc Lamps. (Here the 6258 300W Xe OZONE FREE is what we used). B) The spectral irradiance of natural sunlight and 300W Xe light with AM1.5g filter.

(2) Light scattering by condensed liquid films on glass reactor surfaces

The solar energy absorbed by the surface (P_{sun}) of HHE NFMs under outdoor conditions and indoor conditions can be calculated by[1]:

$$P_{sun} = \int_0^\infty [\varepsilon_s(\lambda, \theta_{sun}) I_{AM1.5}(\lambda)]\, d\lambda$$

where $\varepsilon_s(\lambda, \theta_{sun})$ represents the absorptance/emittance of the surface of HHE NFMs, which is PTC layer, and $I_{AM1.5}(\lambda)$ represents the AM1.5 solar irradiance. In the indoor hydrogen production tests, the HHE NFMs were encapsulated within a **quartz glass reactor** to maintain a sealed environment and prevent hydrogen leakage. Theoretically, the P_{sun} calculation should be based on the standard simulated solar radiation of 1000 W/m². However, under indoor conditions, the temperature of the reactor's top glass window and sidewalls remained close to ambient temperature (~25°C), and water vapor released during evaporation condensed on these surfaces, forming liquid droplets beneath the glass window. Optical characterization reveals that these condensate droplets induce notable light scattering and absorption. Consequently, the actual photon flux reaching the PTC layer was reduced. To validate this conclusion, we set the simulated solar irradiance to 1000 W/m² (as shown in the Fig. S51A). Under illumination, a liquid film rapidly formed and persisted on the underside of the quartz glass cover plate (Fig. S51B). To evaluate the impact of this liquid film on light intensity, a quartz plate with an analogous liquid film was positioned above a radiometer at the same height as the reactor's quartz cover plate. The results demonstrated a significant irradiance reduction due to light scattering by the liquid film, decreasing from 1000 W/m² to **745 W/m²** (Fig. S51C). Consequently, the effective solar irradiance absorbed by the HHE NFMs surface within the reactor under indoor conditions ($P_{sun,\ indoor}$) was calculated as **534.1 W/m²**. The presence of the liquid film in the enclosed reactor thus reduced the solar irradiance absorbed by the PTC layer, leading to both (i) lower surface temperature compared to the open-environment measurement under indoor conditions and (ii) lower photoexcitation of photocatalyst to drive water splitting.

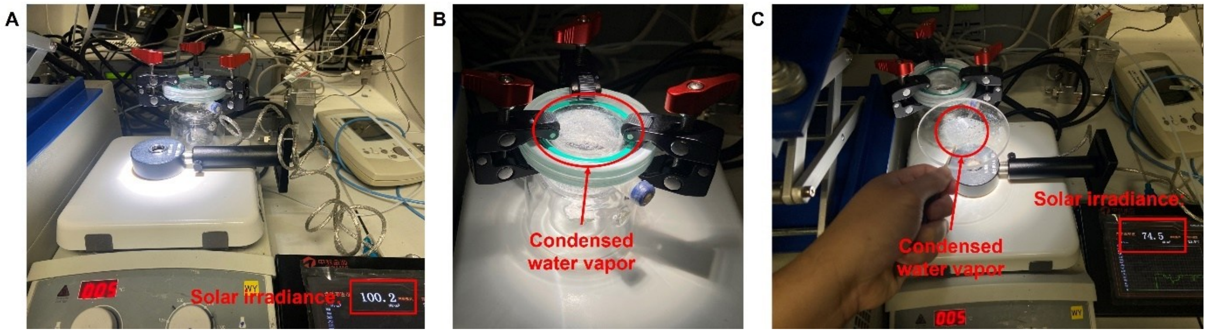

Fig. S51| A) The test figure of setting the indoor Xe lamp to 1000W/m^2 with AM1.5G filter. B) Photo of H_2 production test under indoor Xe lamp and the condensed water vaper under

the quartz glass. C) The test photo of solar irradiance when the quartz glass is covered by condensed water vapor.

However, in the outdoor air-to-H_2 system, the Supplementary Video 1 reveals that although a thin liquid film initially forms on the quartz glass surface, it rapidly dissipates under natural solar irradiation, resulting in negligible light-scattering effects. Under normal outdoor midday sunlight (1000 W/m^2), the power received by the PTC layer surface is about 713 W/m^2, which is larger than indoor condition of 534.1 W/m^2. Even under an average outdoor solar irradiance of 786 W/m^2, the effective irradiance absorbed by the PTC layer ($P_{sun,\ outdoor}$) reaches 569.1 W/m^2. This demonstrates that $P_{sun,\ outdoor}$ (569.1 W/m^2) > $P_{sun,\ indoor}$ (534.1 W/m^2). The superior solar energy absorption capacity of HHE NFMs under outdoor conditions is therefore confirmed.

Besides, most researchers intuitively assume that higher wind speeds in outdoor environments enhance convective heat transfer. However, in our outdoor air-to-H_2 system, the reactor body is constructed using aluminum (Al). This Al reactor undergoes slight heating under sunlight, resulting a temperature rise to slightly above ambient temperature. Therefore, it can be inferred that the solar radiation absorbed by the reactor body is offset by external natural convective cooling, with negligible impact on the surface temperature of PTC layer.

Consequently, in the outdoor air-to-H_2 system, the HHE NFMs exhibit both higher solar energy absorption and higher surface temperature of PTC layer (Fig. 4d). As demonstrated by our DFT calculations (Fig. 3h), the increased reaction temperature within the biphasic system lowers the energy barrier for H_2 formation, thereby accelerating the hydrogen evolution rate. Thus, from a thermodynamic perspective, the enhanced surface temperature of HHE NFMs under outdoor conditions contributes significantly to the improvement in hydrogen production rates compare to the indoor conditions.

Note S3 The energy balance calculation of PTC layer under different conditions

1) Indoor energy balance on the surface of HHE NFMs

In the indoor surface temperature test, the HHE NFMs was placed on an electronic balance for surface temperature test and water evaporation test, where the electronic balance has the glass wall on the surrounding to protect the HHE NFMs from being affected by the wind. Therefore, as we shown in manuscript Fig. 3C, the surface temperature of PTC layer increased from ~25 °C to ~58 °C within 10 min and finally arrived around 68 °C. Therefore, in energy balance calculation, we choose to use the surface temperature of 58 °C in the calculation, as under this temperature the evaporation rate is the highest. Therefore, the P_{sun} can be calculated by:

$$P_{sun}=\int_0^\infty [\varepsilon_s(\lambda,\theta_{sun})I_{AM1.5}(\lambda)]\,d\lambda$$

Here, P_{sun} is 718.9 W/m^2.

Under indoor condition, the passive cooling to the universe can be ignored. Therefore, under the indoor condition,

$$P_{rad}(T_s) = \varepsilon \times \sigma \times (T_s^4 - T_{amb}^4) = 221.8 \text{ W/m}^2$$

The calculation of non-radiative cooling follows:

$$P_{non-radiative,indoor} = h_{non-radiative,indoor}\left(T_{s,indoor} - T_{amb,indoor}\right)$$

Here, due to the surface area of HHE NFMs under indoor condition is 4×10^{-4} m^2, the $h_{non-radiative,indoor}$ = 10.2W/(m^2·K). Therefore, $P_{non-radiative,indoor}$ = 336.6 W/m^2.

According to the original manuscript, the evaporation rate under 1 sun illumination in the open indoor environment is approximately equivalent to that in the outdoor environment.

$$P_{evaperation} = h_{evaperation}\dot{m} = 187 \text{ W/m}^2.$$

Therefore, under indoor open area,

$$P_{evaperation} + P_{non-radiative,indoor} + P_{rad,indoor} + P_{reaction,indoor} = 746.4 \text{ W/m}^2.$$

This value of 746.4 W/m^2 closely aligns with P_{sun}=718.9 W/m^2. The extra energy loss may come from slight inaccuracy of the measured indoor temperature, because under the simulated sunlight, the space inside the electronic balance also heats up, so $P_{non-radiative,indoor}$ may be overestimated here. **Therefore, the calculation results validate the energy balance at the PTC layer surface under indoor open area condition.**

2) Outdoor energy balance on the surface of HHE NFMs under open area

In the outdoor experiments, the HHE NFMs were placed in an **open plastic enclosure (without quartz glass shielding) for surface temperature test**, effectively operating under windless conditions due to the enclosure's walls. As shown in the temperature distribution data in the original manuscript, the maximum surface temperature reached 82°C. While this peak temperature was used in the initial radiative cooling power calculations in the comment, we acknowledge that using the average surface temperature of 76°C would yield a more representative energy balance. Furthermore, the solar irradiance during peak temperature conditions (noon) was measured as ~970 W/m² (close to 1 sun intensity), whereas the value of 760 W/m² cited in the original manuscript represents the daily average irradiance between 10:00 AM and 4:00 PM. Regarding the radiative cooling power calculation, after thorough literature review and validation, we have refined the energy balance framework. Under outdoor conditions, as shown in Fig. S52, the energy conservation equation for the HHE NFMs surface should be expressed as:

$$P_{sun}=P_{rad}(T_s)-P_{atm}(T_{amb})+P_{non-radiative}+P_{evaperation}+P_{reaction}$$

where P_{sun}, $P_{rad}(T_s)$, $P_{atm}(T_{amb})$, $P_{non\text{-}radiative}$, $P_{evaperation}$ and $P_{reaction}$ is the absorbed solar power, radiated power by the PTC layer, absorbed radiation power from the atmosphere, non-radiative cooling power, power absorbed by water evaporation process, and power for hydrogen production. T_s and T_{amb} is the temperature of PTC layer surface exposed to the sky and ambient temperature, which is 76°C and 35°C, respectively.

When the PTC layer is exposed to the sunlight, the P_{sun} can be calculated by[1]:

$$P_{sun}=\int_0^{\infty}[\varepsilon_s(\lambda,\theta_{sun})I_{AM1.5}(\lambda)]\,d\lambda$$

where $I_{\mathrm{AM1.5}}(\lambda)$ represents the AM1.5 solar irradiance. When the clouds move to not block the sun, the light intensity is about 1sun, and the actual value is about 970 W/m^2. Therefore, the P_{sun}=697.33 W/m^2.

Due to the high infrared emissivity of PTC layer, it can spontaneously radiate heat through the atmospheric window. Under the condition of zero humidity, no clouds and clean air atmosphere (no aerosol, etc.), the theoretical value of surface radiation ($P_{rad,ideal}(T_s)$) to the sky should be calculated according to the following equation:[2]

$$P_{rad,ideal}(T_s)=\int_{\Omega}\int_0^{\infty}[\varepsilon_s(\lambda,\theta)I_{BB}(\lambda,T_s)]\,d\lambda cos\theta d\Omega$$

where $\int_{\cap} d\Omega = \int_0^{\pi/2} sin\theta d\theta \int_0^{2\pi} d\phi$ is the hemisphere angular integral, and $\varepsilon_s(\lambda, \theta)$ is the spectral and angular emissivity of the PTC layer. $I_{BB}(\lambda, T_s) = \frac{2hc^2}{\lambda^5}\frac{1}{e^{\frac{hc}{\lambda k_B T_s}}-1}$ indicates the spectral radiance of a blackbody, where h is Planck's constant, c is the speed of light in vacuum, and k_B is the Boltzmann constant.

In this theoretical condition, the theoretical maximum radiation power of our PTC layer is:

$$P_{rad,ideal}(T_s) = 499.6 \text{ W/m}^2.$$

However, in practice, the atmospheric humidity, aerosol content, and cloud coverage significantly influence radiative cooling power. First, water vapor exhibits strong absorption of infrared radiation beyond wavelength of 16 μm.[3] As humidity increases, the 16–22 μm radiative window becomes substantially attenuated or even closed. Specifically, when environmental RH rises from 20% to 100%, the peak atmospheric transmittance in the primary radiative window (8–13 μm) decreases from 90% to 60%. Compared to dry conditions, most secondary radiative windows either disappear (e.g., 16–22 μm) or narrow (e.g., 2.5–5 μm) under high humidity.[2,4] Additionally, atmospheric aerosols (suspended solid/liquid particles) induce Rayleigh and Mie scattering across both solar and thermal infrared spectra.[5,6] Higher humidity exacerbates aerosol scattering, leading to increased infrared attenuation. In Hong Kong, a coastal humid city where our outdoor experiments were conducted, summer aerosols are particularly pronounced. At typical experimental conditions (average RH = 60~80%), aerosol scattering suppresses the atmospheric transmittance in the primary window by up to 20%.[3]

To conservatively account for the combined effects of humidity and aerosols on radiative cooling under cloudless skies, we introduced a correction factor R in our calculation.[3] Generally, the smaller the R is, the greater the impact of the environment on the radiation cooling effect. In order to simplify the calculation and comprehensively consider the influence of RH (60%-80% RH) and aerosol effect in the air, we adopted a conservative value of $R = 0.8$ in our calculations. Therefore, the actual radiative cooling power can thus be expressed as:

$$P_{rad,Ts} = R \times P_{rad,ideal} = 0.8 \times 499.6 = 399.68 \text{ W/m}^2.$$

The power calculation of PTC layer absorbing radiation from the environment without cloud occlusion follows:[2]

$$P_{atm,noncloud}(T_{amb}) = \int_{\cap} \varepsilon_s(\lambda, \theta) \int_0^{\infty} [\varepsilon_{atm}(\lambda, \theta) I_{BB}(\lambda, T_{abm})] \, d\lambda cos\theta d\Omega$$

where $\varepsilon_{atm}(\lambda,\theta) = 1 - t(\lambda)^{1/cos\theta}$, is the emissivity of atmosphere, $t(\lambda)$ is the transmittance of the atmosphere.

Furthermore, the presence of clouds significantly impacts radiative cooling performance.[7] Clouds often behave as near-blackbody emitters in the mid-infrared spectrum, becoming opaque to outgoing thermal radiation. This enhances atmospheric re-radiation and blocks heat dissipation from the Earth's surface, thereby reducing radiative cooling power. As demonstrated by Zhao et al.[8], the decline in radiative cooling power is proportional to the increased cloud cover duration as compared to clear-sky conditions. Besides, the presence of cloud layers leads to increased atmospheric re-radiation, resulting in a higher $P_{atm}(T_{amb})$. Taking June 2024 as an example (when outdoor experiments were conducted), Hong Kong experienced predominantly cloudy conditions with an average cloud cover of 86% (f_c) (The cloud cover data comes from Hong Kong Observatory). Even on clear days, abundant floating cloud layers persisted, which reduced the radiative cooling power of the PTC layer and increased the environmental radiation incident on the PTC. Here, we do not account for the cooling power reduction caused by cloud cover but focus solely on its effect on enhancing P_{atm}.

In the case of cloud cover, the received ambient radiation $P_{atm,\ undercloud}$ is:[3]

$$P_{atm,undercloud} = \int_{\cap} \varepsilon_s(\lambda,\theta) \int_0^{\infty} [\varepsilon_{cloud}(\lambda,\theta) I_{BB}(\lambda,T_{amb}) \tau_{atm}(\lambda,RH,T_{amb},\theta)]\, d\lambda cos\theta d\Omega$$

where $\varepsilon_{cloud}(\lambda,\theta)$ is the is the emissivity of cloud, here we take $\varepsilon_{cloud}(\lambda,\theta) = 1$. $\tau_{atm}(\lambda,RH,T_{amb},\theta)$ is the emissivity of atmosphere under cloud. Therefore,

$$P_{atm}(T_{amb}) = f_c P_{atm,undercloud} + (1 - f_c) P_{atm,noncloud} = 257.3 \text{ W/m}^2.$$

The calculation of non-radiative cooling follows:

$$P_{non-radiative} = h_{non-radiative}(T_s - T_{amb})$$

Where $h_{non-radiative}$ represents non-radiative heat transfer coefficient. In this setup, thermal insulation materials were applied to the sidewalls, and a windbreak layer was implemented to minimize additional heat loss caused by wind. Consequently, heat transfer in this configuration is dominated by natural convection. Following established natural convection heat transfer correlations, the calculated non-radiative heat transfer coefficient is $h_{non-radiative} = 8.0\, W/(m^2 \cdot K)$. Therefore,

$$P_{non-radiative} = h_{non-radiative}(T_s - T_{amb}) = 328 \text{ W/m}^2$$

According to the original manuscript, the evaporation rate under 1 sun illumination in the open indoor environment is approximately equivalent to that in the outdoor environment.

$$P_{evaperation} = h_{evaperation}\dot{m} = 187 \text{ W/m}^2.$$

It is mentioned in the draft that under outdoor conditions, the STH in sunny days is 0.1%. Therefore, $P_{reaction} = STH \cdot P_{AM1.5} = 1$ W/m^2.

As a result,

$$P_{rad}(T_s) - P_{atm}(T_{amb}) + P_{non-radiative} + P_{evaperation} + P_{reaction} = 658.38 \text{ W/m}^2$$

This value of 658.68 W/m^2 closely aligns with P_{sun}=697.33 W/m^2. The residual energy discrepancy likely arises from unaccounted energy losses. **This calculation results validates the energy balance at the PTC layer surface under outdoor open area condition.**

3) Outdoor energy balance on the surface of HHE NFMs in the large scale reactor

When the HHE NFMs were placed in the outdoor large scale reactor, **the quartz glass window can block most of the radiative cooling into the universe under sunny day**, due to its low transmittance in atmospheric radiation window. However, its transmittance is extremely high in the UV and visible light range, so the light intensity here is still calculated based on natural light:

$$P_{sun} = \int_0^{\infty} [\varepsilon_s(\lambda, \theta_{sun}) I_{AM1.5}(\lambda)]\, d\lambda$$

Here, we take the midday outdoor natural sunlight as example, because the surface temperature was tested under midday. So, P_{sun} =697.33 W/m^2.

When HHE NFMs are enclosed in a reactor, the calculation of their radiative heat dissipation should follow:

$$P_{rad}(T_s) = \varepsilon \times \sigma \times \left(T_{s,outdoor}^4 - T_{amb,outdoor}^4\right)$$

Here, as it is difficult to get the temperature inside the reactor, the surface temperature of PTC layer and ambient temperature also taken as 76°C and 35 °C. Therefore,

$$P_{rad}(T_s) = 314.3 \text{ W/m}^2.$$

Most researchers intuitively assume that higher wind speeds in outdoor environments enhance convective heat transfer. However, in our outdoor air-to-H_2 system, the reactor body is constructed of aluminum (Al). This Al reactor undergoes slight heating under sunlight (moderate temperature rises, slightly above ambient temperature, here taken as 35°C). Therefore, it can be inferred that the solar radiation absorbed by the reactor body is offset by external natural convective cooling, with negligible impact on the surface temperature of PTC layer. For energy analysis focusing

solely on the PTC layer, since the HHE NFMs are enclosed within reactors in both indoor and outdoor configurations, natural convective heat transfer occurs at the PTC layer surface in all cases.

$$P_{non-radiative} = h_{non-radiative}(T_s - T_{amb}) = 328 \text{ W/m}^2.$$

Furthermore, when the HHE NFM was placed in a reactor (either under outdoor conditions or indoor conditions), the evaporation rate of the HHE NFM was greatly reduced in a closed environment as shown in Fig. S34. The obviously lower evaporation rate in the closed system is mainly because (i) the significantly higher relative humidity in the closed system inhibits evaporation,[9,10] and (ii) the convection in the closed system is much less than that in the open system.[11,12]

Therefore, the evaporation power was calculated as:

$$P_{evaperation} = h_{evaperation}\dot{m} = 29.6 \text{ W/m}^2.$$

It is mentioned in the manuscript that under outdoor conditions, the STH in sunny days is 0.1%. Therefore,

$$P_{reaction} = STH \cdot P_{AM1.5} = 1 \text{ W/m}^2.$$

Therefore, in outdoor large scale reactor,

$$P_{evaperation} + P_{non-radiative,indoor} + P_{rad,indoor} + P_{reaction,indoor} = 675.8 \text{ W/m}^2$$

This value of 675.8 W/m^2 closely aligns with P_{sun}=697.3 W/m^2 within the outdoor large scale reactor. The residual energy discrepancy likely arises from unaccounted energy losses.

Therefore, based on our calculations, HHE NEMs verify energy conservation in both outdoor and indoor conditions.

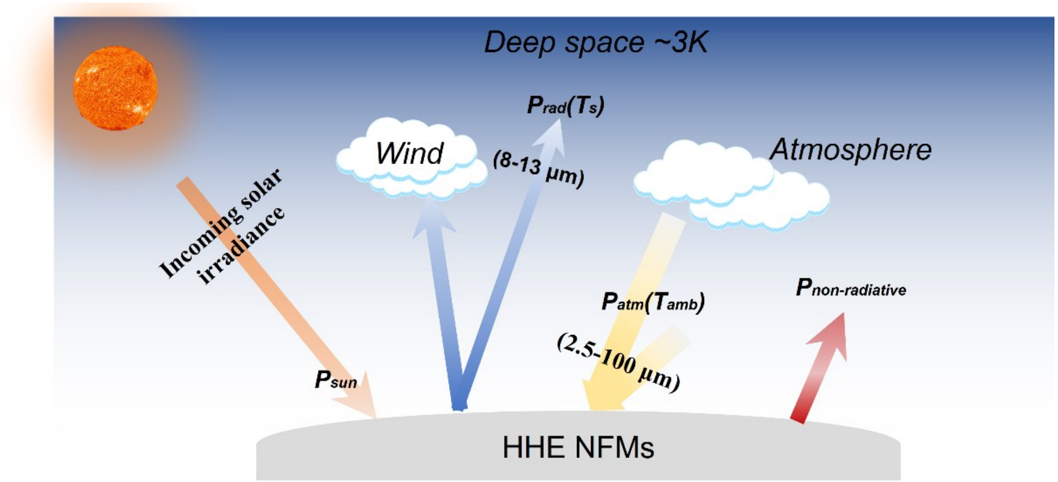

Fig. S52 Schematic diagram and heat transfer processes of PTC layer.

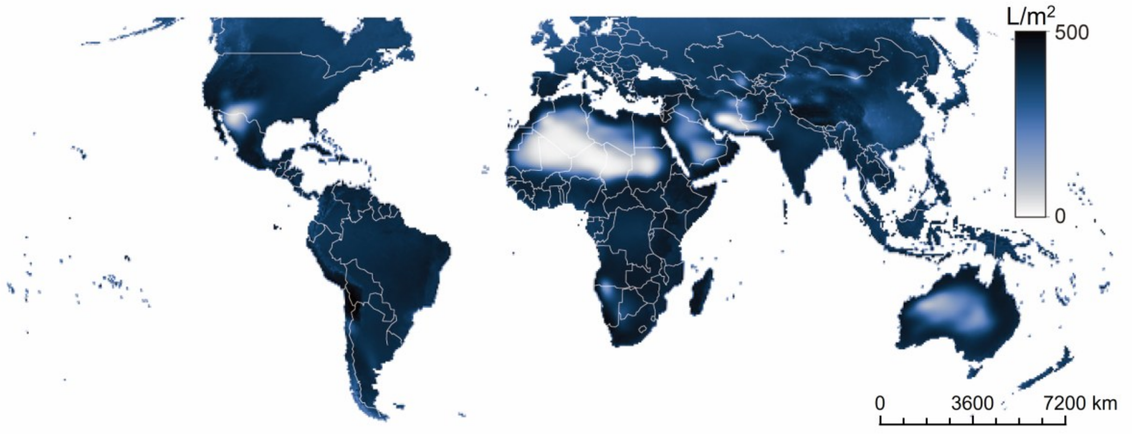

Fig. S53|Potential of H_2 production from HHE systems across terrestrial regions from 60°N to 60°S latitude throughout the year.

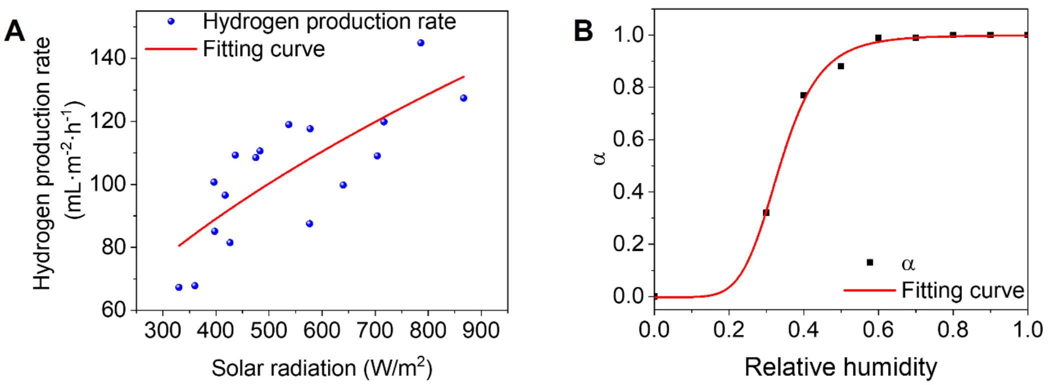

Fig. S54| A) Relationship curve between average solar radiation and average hydrogen production rate. B) The relationship between RH and the water adsorption saturation (*α*) of HHE NFMs

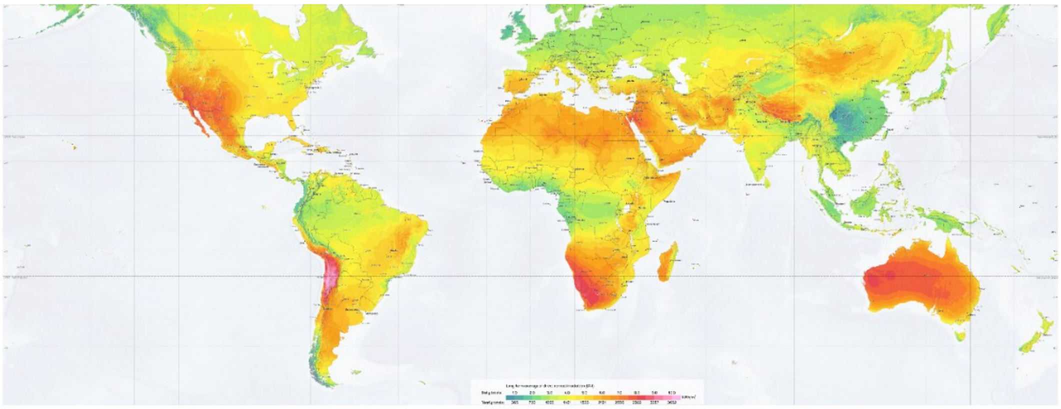

Fig. S55| Direct solar irradiation all over the world. Source: World Bank Group, accessed on [05.2024], https://globalsolaratlas.info

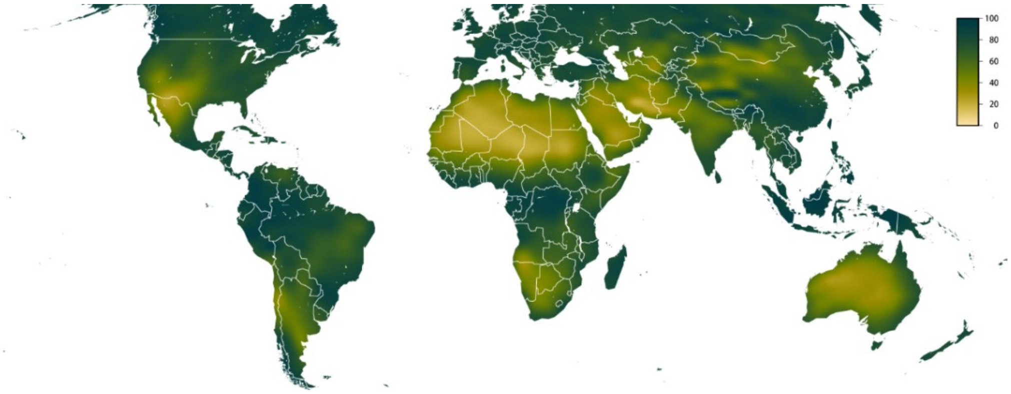

Fig. S56| Global nighttime average humidity distribution. Source: Asia-Paific Data-research Center, Reanalysis Data, NCEP Reanalysis, accessed on [05.2024], http://apdrc.soest.hawaii.edu/las/v6/dataset?catitem=16801

Table S1 The comparison of air-to-H_2 system with other photocatalytic H_2 production technologies

Photocatalyst	STH Efficiency	Solar energy required to produce one mol of hydrogen (J/mol)	Feedstock	Light source	Ref
Pt/TiO_2	0.10%	2.3×10^{8}	Vaper harvested for the air	Natural sunlight	This work
RhCrOx-Al:$SrTiO_3$	0.09%	2.63×10^{8}	Vapor evaporated from pure liquid water	Natural sunlight	[13]
CoOOH/Rh loaded $SrTiO_3$:Al coated with TiO_x	0.4%	5.93×10^{7}	Pure vapor feeding	300 W Xe lamp AM 1.5G	[14]
Pt/TiO_2	0.002%	1.19×10^{10}	Liquid seawater	500 W Hg lamp	[15]
Ru-modified $SrTiO_3$: La, Rh/Au colloid (40 wt%)/$BiVO_4$:Mo	0.1%	2.37×10^{8}	Bulk pure water	300 W Xe lamp	[16]
g-C_3N_4/ITO/Co–$BiVO_4$	0.028%	8.46×10^{8}	Bulk pure water	300 W Xe lamp AM 1.5 G	[17]
Rh/CrO_x Au/CoO_x modified $PbTiO_3/BiVO_4$	0.053%	4.47×10^{8}	Bulk pure water containing Fe^{2+}/Fe^{3+}	300 W Xe lamp $\lambda >$ 420 nm	[18]

$BiVO_4/Au/CdS$	0.054%	4.39×10^{8}	Bulk pure water	300W Xe lamp AM 1.5 G	19
$CoOx/CdTe/V\text{-}In_2S_3$	1.31%	1.87×10^{7}	Bulk pure water	300 W Xe lamp λ≥300 nm	20
$Pt/CoOx/ZnIn_2S_4/WO_3$	1.52%	1.56×10^{7}	Bulk pure water	300 W Xe lamp AM 1.5 G	21
Cu/TiO_2	0.002%	1.19×10^{10}	Methanol aqueous solution	Natural sunlight	22
Cu/TiO_2	0.074%	3.2×10^{8}	Methanol aqueous solution	300W Xe AM1.5G	22
PdPSA-CdS	0.1%	2.3×10^{8}	Ethanol aqueous solution	365 mW LED λ=420 nm	23
$Pt\text{-}IrO_2/3D\text{-}g\text{-}C_3N_4$	0.06%	3.95×10^{8}	Bulk pure water	300 W Xe lamp λ≥420 nm	24
Ni–Ag/CN_x	0.02%	1.19×10^{9}	Ethanol aqueous solution	300 W Xe lamp AM 1.5 G	25

Table S2 The comparisons of photocatalytic air-to-H_2 system.

Photocatalyst	Feedstock	STH efficiency	quantum efficiency	System scale	Light source	Ref.
Pt/TiO_2	Moisture harvested from the air	**0.10%**	NA.	**2.5×10^{-1} m^2**	Natural sunlight	This work
TiO_x-coated CoOOH/Cr_2O_3/Rh/$SrTiO_3$:Al	Moisture harvested from the air	0.08%	0.11%	1.075×10^{-3} m^2	Natural sunlight and UV LED (380 nm)	26
TiO_x-coated CoOOH/Cr_2O_3/Rh/$SrTiO_3$:Al	Moisture harvested from the air	0.12%	0.11%	1.075×10^{-3} m^2	300W Xe lamp（100 mW cm^{-2}） and UV LED (380 nm)	26
Pt-TiO_2	Moisture harvested from the air (with polyethylene glycol as sacrificial agent)	0.0859 %	NA.	2.83×10^{-3} m^2	Natural sunlight	27
Pt-TiO_2	Moisture harvested from the air (with ethylene	0.14%	NA.	5×10^{-2} m^2	Simulated sunlight（100 mW cm^{-2}）	28

	glycol as sacrificial agent)					
$SrTiO_3$:Al-$RhCrO_x$ -CoO_y	Moisture from the air	NA.	0.34%	1.35×10^{-4} m^2	UV LED (365 nm, 14.27mW cm^2.)	29
RhCrOx-Al:$SrTiO_3$	Moisture evaporated from pure liquid water	0.09%	NA.	2.5×10^{-3} m^2	Natural sunlight	13
Py-HMPA@Pt	Moisture evaporated from pure water	0.064%	7.9%	3.14×10^{-4} m^2	300 W Xenon lamp with filter (λ > 420 n m, 100 mW c m^2), UV LED (420 nm)	30
CoOOH/Rh loaded $SrTiO_3$:Al coated with TiO_x	Pure vapor feeding	0.4%	NA.	4×10^{-4} m^2	AM 1.5G simulated sunlight (~100 mW cm^2)	14
Pt-CN	Pure vapor feeding (flow rate 2 mL min^{-1})	0.26%	NA	4×10^{-4} m^2	Simulated sunlight (100 mW cm^{-2})	31

Table S3. Material properties and parameters used in the simulations

Nomenclature	Value
Molecular weight of dry air, M_a	0.028 kg·mol^{-1}
Dry air thermal conductivity, k_a	0.025 W·m^{-1}·K^{-1}
Dry air heat capacity, $C_{p,a}$	1.006×10^{-5} J·kg^{-1}·K^{-1}
Water thermal conductivity, k_w	0.59 W·m^{-1}·K^{-1}
Water heat capacity, $C_{p,w}$	4.182×10^{3} J·kg^{-1}·K^{-1}
Water density, ρ_w	998.2 kg·m^{-3}
Vapor thermal conductivity, k_v	0.026 W·m^{-1}·K^{-1}
Vapor heat capacity, $C_{p,v}$	2.062×10^{3} J·kg^{-1}·K^{-1}
Porosity of HHE NFMs, φ_H	0.93
Density of HHE NFMs	590 kg·m^{-3}
Thermal conductivity of HHE NFMs, k_H	0.0525 W·m^{-1}·K^{-1}
Heat capacity of HHE NFMs, $C_{p,H}$	838 J·kg^{-1}·K^{-1}
Latent heat of evaporation, H_v	2.454×10^{6} J·kg^{-1}

Table S4. Initial and boundary conditions in the simulations

Initial/boundary condition	Value
Initial ambient pressure, P_0	1.01325×10^{5} Pa
Initial ambient air temperature, T_0	298.15 K
Initial ambient RH	0.5
Initial water uptake capacity of HHE NFMs	0.90 g/g
Initial velocity within the flow field	0 m/s
Radiation heat flux absorbed by PTC layer, Q_R	800 W/m^{-2}
The pressure of water vapor outlet	1.01325×10^{5} Pa
Heat outflow at boundary	Natural convection heat transfer

Supplementary movie 1

This movie shows the daytime H_2 production of large-scale air-to-hydrogen system.

Supplementary movie 2

This movie demonstrates sustainable hydrogen production of our prototype powers ~1000 LEDs. Here, we connected the reactor after sunlight with the circulation pump, drying tube and fuel cell to form a gas circuit. Then, the electrodes of the fuel cell were connected with the LED bulbs to release electric energy.

References

1 Yin, Y. *et al.* A Colored Temperature‐Adaptive Cloak for Year‐Round Building Energy Saving. *Advanced Energy Materials* (2024). https://doi.org:10.1002/aenm.202402202

2 Raman, A. P., Anoma, M. A., Zhu, L., Rephaeli, E. & Fan, S. Passive radiative cooling below ambient air temperature under direct sunlight. *Nature* **515**, 540-544 (2014). https://doi.org:10.1038/nature13883

3 Huang, J., Lin, C., Li, Y. & Huang, B. Effects of humidity, aerosol, and cloud on subambient radiative cooling. *International Journal of Heat and Mass Transfer* **186** (2022). https://doi.org:10.1016/j.ijheatmasstransfer.2021.122438

4 Eldridge, R. G. Water vapor absorption of visible and near infrared radiation.

5 He, Q., Li, C., Mao, J., Lau, A. K. H. & Chu, D. A. Analysis of aerosol vertical distribution and variability in Hong Kong. *Journal of Geophysical Research: Atmospheres* **113** (2008). https://doi.org:10.1029/2008jd009778

6 Mishra, A. K., Koren, I. & Rudich, Y. Effect of aerosol vertical distribution on aerosol-radiation interaction: A theoretical prospect. *Heliyon* **1**, e00036 (2015). https://doi.org:https://doi.org/10.1016/j.heliyon.2015.e00036

7 Han, D., Ng, B. F. & Wan, M. P. Preliminary study of passive radiative cooling under Singapore's tropical climate. *Solar Energy Materials and Solar Cells* **206**, 110270 (2020). https://doi.org:https://doi.org/10.1016/j.solmat.2019.110270

8 Zhao, B., Hu, M., Ao, X. & Pei, G. Performance evaluation of daytime radiative cooling under different clear sky conditions. *Applied Thermal Engineering* **155**, 660-666 (2019). https://doi.org:https://doi.org/10.1016/j.applthermaleng.2019.04.028

9 Batishcheva, K. *Evaporation time of water droplets in an isolated chamber*. Vol. 2212 (2020).

10 Cioulachtjian, S., Launay, S., Boddaert, S. & Lallemand, M. Experimental investigation of water drop evaporation under moist air or saturated vapour conditions. *International Journal of Thermal Sciences* **49**, 859-866 (2010). https://doi.org:https://doi.org/10.1016/j.ijthermalsci.2009.12.014

11 Xu, Z. *et al.* Ultrahigh-efficiency desalination via a thermally-localized multistage solar still. *Energy & Environmental Science* **13**, 830-839 (2020). https://doi.org:10.1039/C9EE04122B

12 Chen, K., Li, L. & Zhang, J. Elucidating differences in solar-driven interfacial evaporation between open and closed systems. *Desalination* **564**, 116791 (2023). https://doi.org:https://doi.org/10.1016/j.desal.2023.116791

13 Pornrungroj, C. *et al.* Hybrid photothermal–photocatalyst sheets for solar-driven overall water splitting coupled to water purification. *Nature Water* **1**, 952-960 (2023). https://doi.org:10.1038/s44221-023-00139-9

14 Suguro, T. *et al.* A hygroscopic nano-membrane coating achieves efficient vapor-fed photocatalytic water splitting. *Nature communications* **13**, 5698 (2022). https://doi.org:10.1038/s41467-022-33439-x

15 Zhang, J. *et al.* Photocatalytic hydrogen production from seawater under full solar spectrum without sacrificial reagents using TiO2 nanoparticles. *Nano Research* **15**, 2013-2022 (2021). https://doi.org:10.1007/s12274-021-3982-y

16 Kato, H., Sasaki, Y., Shirakura, N. & Kudo, A. Synthesis of highly active rhodium-doped SrTiO3 powders in Z-scheme systems for visible-light-driven photocatalytic overall water splitting. *Journal of Materials Chemistry A* **1** (2013). https://doi.org:10.1039/c3ta12803b

17 Dai, D. *et al.* g-C3N4/ITO/Co-BiVO4 Z-scheme composite for solar overall water splitting. *Chemical Engineering Journal* **433** (2022). https://doi.org:10.1016/j.cej.2021.134476

18 Kang, Y. *et al.* Ferroelectric polarization enabled spatially selective adsorption of redox mediators to promote Z-scheme photocatalytic overall water splitting. *Joule* **6**, 1876-1886 (2022). https://doi.org:10.1016/j.joule.2022.06.017

19 Xu, X. *et al.* Refined Z-scheme charge transfer in facet-selective BiVO4/Au/CdS heterostructure for solar overall water splitting. *International Journal of Hydrogen Energy* **46**, 8531-8538 (2021). https://doi.org:10.1016/j.ijhydene.2020.12.047

20 Zhang, Y. *et al.* Internal quantum efficiency higher than 100% achieved by combining doping and quantum effects for photocatalytic overall water splitting. *Nature Energy* **8**, 504-514 (2023). https://doi.org:10.1038/s41560-023-01242-7

21 Wang, Y. *et al.* Sulfur‐Deficient ZnIn2S4/Oxygen‐Deficient WO3 Hybrids with Carbon Layer Bridges as a Novel Photothermal/Photocatalytic Integrated System for Z‐Scheme Overall Water Splitting. *Advanced Energy Materials* **11** (2021). https://doi.org:10.1002/aenm.202102452

22 Lee, W. H. *et al.* Floatable photocatalytic hydrogel nanocomposites for large-scale solar hydrogen production. *Nature nanotechnology* **18**, 754-762 (2023). https://doi.org:10.1038/s41565-023-01385-4

23 Zhou, P. *et al.* Partially reduced Pd single atoms on CdS nanorods enable photocatalytic reforming of ethanol into high value-added multicarbon compound. *Chem* **7**, 1033-1049 (2021). https://doi.org:10.1016/j.chempr.2021.01.007

24 Chen, X. *et al.* Three-dimensional porous g-C3N4 for highly efficient photocatalytic overall water splitting. *Nano Energy* **59**, 644-650 (2019). https://doi.org:10.1016/j.nanoen.2019.03.010

25 Chai, Z., Mattsson, A., Tesfamhret, Y., Österlund, L. & Zhu, J. Ni–Ag Nanostructure-Modified Graphitic Carbon Nitride for Enhanced Performance of Solar-Driven Hydrogen Production from Ethanol. *ACS Applied Energy Materials* **3**, 10131-10138 (2020). https://doi.org:10.1021/acsaem.0c01838

26 Yang, W. *et al.* Synergistic Integration of Atmospheric Water Harvesting and Solar‐Driven Hydrogen Production via Multifunctional Hygroscopic‐Photocatalytic Hydrogel Nanocomposite. *Advanced Functional Materials* (2025). https://doi.org:10.1002/adfm.202512738

27 Huang, L. *et al.* Solar-driven hydrogen production based on moisture adsorption-desorption cycle. *Nano Energy* **128** (2024). https://doi.org:10.1016/j.nanoen.2024.109879

28 Liu, P. *et al.* Scalable hydrogen production by harvesting moisture from the air under natural sunlight. *Chemical Engineering Journal* **510** (2025). https://doi.org:10.1016/j.cej.2025.161832

29 Shearer, C. J., Hisatomi, T., Domen, K. & Metha, G. F. Gas phase photocatalytic water splitting of moisture in ambient air: Toward reagent-free hydrogen production. *Journal of Photochemistry and Photobiology A: Chemistry* **401**, 112757 (2020). https://doi.org:10.1016/j.jphotochem.2020.112757

30 Liu, Y. *et al.* One-dimensional covalent organic frameworks with atmospheric water harvesting for photocatalytic hydrogen evolution from water vapor. *Applied Catalysis B: Environmental* **338** (2023). https://doi.org:10.1016/j.apcatb.2023.123074

31 He, L. *et al.* A Hybrid Photocatalytic System Splits Atmospheric Water to Produce Hydrogen. *Advanced Functional Materials* **34** (2024). https://doi.org:10.1002/adfm.202313058
