# Supplementary material for: Electricity-free hydrogen production from the air
Source: Nat Commun. 2026 Feb 4;17:1445. doi: 10.1038/s41467-025-67511-z (PMC12887035; doi:10.1038/s41467-025-67511-z)
Supplement: Supplementary file 2 — Description of Additional Supplementary Files [file 41467_2025_67511_MOESM2_ESM.pdf]

Description of Additional Supplementary Files

Supplementary Data 1: This data contents the DFT calculation structures.

Supplementary Movie 1: This movie shows the daytime H2 production of large-scale air-to-hydrogen system.

Supplementary Movie 2: This movie demonstrates sustainable hydrogen production of our prototype powers ~1000 LEDs. Here, we connected the reactor after sunlight with the circulation pump, drying tube and fuel cell to form a gas circuit. Then, the electrodes of the fuel cell were connected with the LED bulbs to release electric energy.
